# Holocentric Karyotype Evolution in *Rhynchospora* Is Marked by Intense Numerical, Structural, and Genome Size Changes

**DOI:** 10.3389/fpls.2020.536507

**Published:** 2020-09-10

**Authors:** Paula Burchardt, Christopher E. Buddenhagen, Marcos L. Gaeta, Murilo D. Souza, André Marques, André L. L. Vanzela

**Affiliations:** ^1^ Laboratório de Citogenética e Diversidade Vegetal, Departamento de Biologia Geral, CCB, Universidade Estadual de Londrina, Londrina, Brazil; ^2^ Forage Science, AgResearch Limited, Hamilton, New Zealand; ^3^ Department of Chromosome Biology, Max Planck Institute for Plant Breeding Research, Cologne, Germany

**Keywords:** C-CMA/DAPI banding, chromosome numbers, DNA C-value, flow cytometry, karyotype diversity

## Abstract

Cyperaceae is a family of Monocotyledons comprised of species with holocentric chromosomes that are associated with intense dysploidy and polyploidy events. Within this family the genus *Rhynchospora* has recently become the focus of several studies that characterize the organization of the holocentric karyotype and genome structures. To broaden our understanding of genome evolution in this genus, representatives of *Rhynchospora* were studied to contrast chromosome features, C-CMA/DAPI band distribution and genome sizes. Here, we carried out a comparative analysis for 35 taxa of *Rhynchospora*, and generated new genome size estimates for 20 taxa. The DNA 2C-values varied up to 22-fold, from 2C = 0.51 pg to 11.32 pg, and chromosome numbers ranged from 2*n* = 4 to 61. At least 37% of our sampling exhibited 2*n* different from the basic number *x* = 5, and chromosome rearrangements were also observed. A large variation in C-CMA/DAPI band accumulation and distribution was observed as well. We show that genome variation in *Rhynchospora* is much larger than previously reported. Phylogenetic analysis showed that most taxa were grouped in clades corresponding to previously described taxonomic sections. Basic chromosome numbers are the same within every section, however, changes appeared in all the clades. Ancestral chromosome number reconstruction revealed *n* = 5 as the most likely ancestral complements, but *n* = 10 appears as a new possibility. Chromosome evolution models point to polyploidy as the major driver of chromosome evolution in *Rhynchospora*, followed by dysploidy. A negative correlation between chromosome size and diploid number open the discussion for holokinetic drive-based genome evolution. This study explores relationships between karyotype differentiation and genome size variation in *Rhynchospora*, and contrasts it against the phylogeny of this holocentric group.

## Introduction 

The size, morphology and composition of chromosomes have been useful parameters for comparing karyotypes of phylogenetically related species, and to resolve some taxonomic conflicts (Guerra, 2000). These features have been widely regarded as drivers of evolutionary processes (Comai, 2005; Doyle et al., 2008), as they are the result of duplication or deletion of entire chromosomes, polyploidy, fission, fusion, and/or chromosome translocation (Greilhuber, 1995; Luceño and Guerra, 1996; Schubert and Lysak, 2011). We focus on karyotype evolution of Rhynchosporeae, a monophyletic clade in the Cyperaceae that presents holocentric chromosomes, a trait that supposedly originated independently in four distinct clades of plants (Melters et al., 2012).

Greilhuber (1995) considered holocentric chromosomes to be a synapomorphy of the Cyperid clade (Thurniaceae, Juncaceae and Cyperaceae), and this characteristic has been accepted for *Rhynchospora* since then (Vanzela et al., 2000; Vanzela and Colaço, 2002; Arguelho et al., 2012). However, Guerra et al. (2019) reported that *Juncus* L., a genus regarded as exclusively holocentric, contains monocentric species. Also recently it was reported that Prionium serratum (Thurniaceae) is also monocentric (Baez et al., 2020). More detailed work in this area is needed to provide insights into the evolution of holocentric chromosomes in Cyperales, such as whether it arose independently in each of the major clades.

In holocentric chromosomes, kinetochore proteins are arranged along the chromosomes and their kinetic activity appears to be distributed along almost the entire chromatid surface (Melters et al., 2012; Heckmann and Houben, 2013). In this case, any fragments produced by chromosome fission/fusion may segregate regularly, making them more likely to be inherited during cell division, and this can lead to increases and decreases in chromosome numbers, giving rise to dysploidy (Mola and Papeschi, 2006; Arguelho et al., 2012). In contrast, fission events in monocentric chromosomes may generate acentric fragments that are unable to segregate normally and are lost during cell division (Carrano and Heddle, 1973; Escudero et al., 2015).

Cyperaceae members are well known for having large chromosome number variation associated with chromosome rearrangements, and *Carex* L. has the highest record of chromosome fission and fusion (Hipp, 2007; Roalson, 2008; Hipp et al., 2009). The increase in chromosome number by polyploidy has also been proposed in genera such as *Eleocharis* R.Br. (Da Silva et al., 2010; Zedek et al., 2010) and *Rhynchospora* Vahl (Vanzela et al., 2000). High chromosome numbers were also found in *Cyperus cyperoides* Kuntze (*n* = 112; Tejavathi, 1988), *C. esculentus* L. (*n* = 104; Sharma, 1970), *Carex hirta* L. (*n* = 56–57; Luceño, 1994), and *Rhynchospora faurieri* Franch. (*n* = 31; Hoshino, 1987). But, the reduction below the probable basic chromosome number (*x* = 5) also happens in Cyperaceae *via* dysploidy, such as *n* = 2 in *Rhynchospora* (Vanzela et al., 1996) and *n* = 3 in both *Eleocharis* (Da Silva et al., 2005) and *Fimbristylis* Vahl (Rath and Patnaik, 1977). Lower numbers have also been reported in other holocentric families, such as Juncaceae (Malheiros et al., 1947), Droseraceae (Kondo et al., 1994) and Convolvulaceae (Pazy and Plitmann, 1987; Pazy and Plitmann, 1994).

A number of cytogenetic studies compared *Rhynchospora* karyotypes (Luceño et al., 1998a; Vanzela and Guerra, 2000; Vanzela et al., 2000; Vanzela et al., 2003; Sousa et al., 2011; Michelan et al., 2012; Cabral et al., 2014; Marques et al., 2015; Ribeiro et al., 2017; Ribeiro et al., 2018), but none compared karyotype diversity with heterochromatin distribution and DNA C-value variation together, encompassing different clades. The genus *Rhynchospora* is the third largest clade in Cyperaceae (Araújo et al., 2012), with ca. 350 species distributed worldwide (Buddenhagen et al., 2017). Chromosome numbers vary from 2*n* = 4 in *R. tenuis* (Vanzela et al., 1996) to 2*n* = 62 in *R. faurieri* (Hoshino, 1987), although 2*n* = 10 is the most common number. Reports suggest a wide diversity with odd and even numbers, like 2*n* = 4, 5, 8, 10, 12, 18, 20, 24, 26, 30, 36, 37, 45, 48, 50, and 58 (Luceño et al., 1998a; Luceño et al., 1998b; Vanzela et al., 2000; Arguelho et al., 2012; Ribeiro et al., 2018), and this high variability can also be observed within a single species, e.g. *R. globosa* (2*n* = 24, 36, 37, 45, 48, 50, and 58).

*Rhynchospora* has been used as a model for detailed studies aiming to characterize holocentric chromosome structure and adaptations taking place in these organisms (Cabral et al., 2014; Marques et al., 2015; Marques et al., 2016; Rocha et al., 2016). A prior study examining the interspecific relationships in a phylogenetic context using cytogenetic data and DNA content has suggested polyploidy as the main driver of karyotype and genome evolution in *Rhynchospora* (Ribeiro et al., 2018). Despite this and the high diversity of the genus, there are few studies approaching phylogenetic relationships with genomes and karyotype data. The most comprehensive analysis of the genus was based on a traditional herbarium based taxonomic study of 211 species (Kükenthal, 1949; Kükenthal, 1950a; Kükenthal, 1950b; Kükenthal, 1951).

In order to perform a comprehensive assessment of evolutionary forces that have a role in *Rhynchospora* karyotype differentiation, the number of Brazilian populations and species was expanded and phylogenetically compared, including never studied species. Efforts were intended to compare intra- and interspecific variations in chromosome number, as well as to estimate DNA C-values and C-CMA/DAPI bands distribution. Data were compared and analyzed in a phylogenetic context, including samples from 14 different taxonomic sections of the genus. Our data provide a window into the group’s intraspecific variation, which helps to support polyploidy and dysploidy as the major drivers of genome and karyotype evolution in *Rhynchospora*, and indicate the importance of wide sampling to include possible inter and intraspecific variations in holocentric karyotypes.

## Materials and Methods

### Plant Material

Living plants of 24 taxa were collected from different localities in Brazil. Plants were grown in pots in the greenhouse of the Center for Biological Sciences at the State University of Londrina and vouchers were deposited in the Herbarium of Londrina State University (FUEL Herbarium). The **Supplementary data 1** for this study contains descriptions of the plant material, chromosome counts and nuclear DNA measurements, including previously published data gathered from the literature. Some chromosome records obtained from previously fixed materials, and that are part of the Laboratório de Citogenética e Diversidade Vegetal (LCDV, UEL, Brazil) collection, were used for comparison purposes and are indicated in tables and figures as “from LCDV” and includes citations.

### Genome Size Estimates

Holoploid genome sizes (2C-values) were assessed for available living specimens of *Rhynchospora*. *Raphanus sativus* L. ‘Saxa’ (2C = 1.11 pg; Doležel et al., 1998), *Solanum lycopersicum* L. ‘Stupicke polni tyckove rane’ (2C = 1.96 pg; Doležel et al., 1992), and *Pisum sativum* L. ‘Ctirad’ (2C = 9.09 pg; Doležel et al., 1998), were each used as internal standards. Young leaves were processed immediately after collection. Fragments (1 cm^2^) of young leaves of the sample and the internal standard were chopped together (Galbraith et al., 1983), for 30 s (Noirot et al., 2005), with a brand-new razor blade in a 60 mm × 10 mm Petri dish containing 125 μl of OTTO-I lysis buffer (Otto, 1990), supplemented with 2.0 mM polyethylene glycol and 50 μg/ml RNAse. To the nuclei suspensions, another 125 μl of OTTO-I lysis buffer was added, and the homogenates were sieved through 25 μm nylon filters into 2.0 microcentrifuge tubes, then centrifuged at 100× g for 5 min. The supernatant of each sample was poured out, and the pellet resuspended and incubated for 5 min in 25 μl of OTTO-I lysis buffer. The suspensions were stained with 375 μl OTTO-II solution (Otto, 1990; Doležel and Göhde, 1995), supplemented with 75 μM propidium iodide, 2.0 mM polyethylene glycol and 50 μg/ml RNAse (Doležel et al., 1992; Meister, 2005). The staining step was performed in the dark for 40 min, followed by filtration through a 25 μm nylon mesh. DNA content of at least 10,000 stained nuclei was determined for each sample using a BD Accuri C6 flow cytometer (BD Biosciences), using three independent DNA estimations of one to three individual plants. Chromosome counts were done for every population sampled, except for *R. pilosa*, for which we used a previously reported chromosome number. Total 2C-values were calculated as sample peak mean/standard peak mean × 2C DNA content of standard (pg). The hypothetical monoploid genome sizes (1C*x*) were calculated by dividing the 2C-values by the ploidy level. Pearson’s correlation test and linear regression analysis were performed in the R statistical software environment with the ggplot2 package (Wickham, 2016). The average chromosome size was calculated for each sample, including genome size and chromosome count data available from our study and literature. Average chromosome size was calculated from genome size data as 2C (Mbp)/2*n* (1 pg = 978 Mbp; Doležel et al., 2003) and plotted together with 2*n* values for comparison.

### Cytogenetic Analyses

Three to five plants of each of the 23 species, comprising 37 populations, provided meristems for cytogenetic analysis. Root tips from greenhouse cultivated plants were pretreated in a solution containing 2 mM 8-hydroxyquinoline at 10°C for 24 h, fixed in 3:1 ethanol:glacial acetic acid (v:v) for 24 h at room temperature, and stored at –20°C. Samples were digested in 2% cellulase plus 20% pectinase (w/v), both Sigma, at 37°C for 45 to 60 min. For conventional staining, digested root tips were washed in distilled water, hydrolyzed in HCl 1 M for 10 min at 60°C, and squashed in a drop of 60% acetic acid. Coverslips were removed after freezing in liquid nitrogen. Samples were stained in 3% Giemsa and mounted in Entellan (Merck).

Chromomycin A_3_ (CMA) and 4-6 diamidino-2-phenyl indole (DAPI) serve to identify heterochromatic blocks by binding to GC- and AT-rich repeats in the genome, respectively. C-CMA/DAPI banding was carried out using fixed root tips digested for 3 to 4 h in a mixture of 2% cellulase and 20% pectinase (w/v), and squashed in a drop of 60% acetic acid. Coverslips were removed in liquid nitrogen and, after air drying for 3 days, samples were treated for C-banding procedure [45% acetic acid at 60°C for 10 min, 5% Ba(OH)_2_ at room temperature for 10 min and 2× SSC, pH 7.0, at 60°C for 1 h and 30 min]. Subsequently, samples were stained with CMA_3_ for 90 min and DAPI for 30 min, as described by Vanzela and Guerra (2000) and mounted in glycerin:McIlvaine buffer (1:1, v:v), pH 7.0, and 2.5 mM MgCl_2_.

Slides were examined using a Leica DM 4500B epifluorescence microscope and images were acquired using a Leica DFC 300FX camera. All the images were optimized for contrast and brightness using Gimp-2.8 and Inkscape 0.92.3 programs on the Linux platform.

### Phylogenetic Comparison

A phylogenetic analysis was undertaken for *Rhynchospora* taxa for which either chromosome number or genome size data is available. This was not intended as a new phylogenetic proposition for the genus, but as a means of complementing the cytogenetic analysis and supporting further comparisons. For this purpose, we extracted plastid sequences from an Illumina short-read target capture data for 35 of 115 available *Rhynchospora* species (Buddenhagen, 2016; Buddenhagen et al., 2016) using the Geneious mapper tool with default parameters available in Geneious version 7.1. Plastome-derived sequences from 35 species of *Rhynchospora* were obtained using the plastomes of *Hypolytrum nemorum* (Vahl) Spreng. (GenBank accession number NC_036036.1) and *Carex neurocarpa* Mack. (GenBank accession number NC_036037.1) as a reference sequence for mapping (see **Supplementary data 2**). A consensus sequence was made for each sampled species, and as criteria, gene regions with more than 15% gaps were stripped, and alignments for gene regions with more than one missing taxon were not used. Filtered regions were aligned with PASTA (Mirarab et al., 2015) and gene alignment files were concatenated in Geneious. Partitions used for tree estimation corresponded to the annotated regions. There were 54 distinct plastid regions, listed in **Supplementary data 3**, used in the final alignment (43,979 bases). The concatenated alignment was then used to estimate maximum likelihood phylogeny using the software IQ-TREE (Nguyen et al., 2015) with 1,000 bootstraps. The consensus tree was edited in FigTree v1.4.2 (http://tree.bio.ed.ac.uk/software/figtree/).

### Ancestral Chromosome Number Reconstruction

To test the best models for chromosome evolution in *Rhynchospora* we applied the RevBayes (Freyman and Höhna, 2018) implementation of the ChromEvol models (Glick and Mayrose, 2014). The program utilizes a model of anagenetic transition rates including gain (fission, dysploidy) or loss (fusion) of chromosomes, polyploidization, and demi-polyploidization. The best fitting model was assessed using the AIC (Glick and Mayrose, 2014). Furthermore, the ancestral states of chromosome number along the branches were estimated using PastML (Ishikawa et al., 2019) applying two prediction methods, Maximum Likelihood (JOINT+F81) and Maximum Parsimony (Accelerated Transformation), respectively. Since these analyses can take only one state per sample, only the lowest chromosome number for each species was used in the case of samples with more than one cytotype, with different chromosome numbers and ploidy levels.

## Results

### Genome Size Variations

**Figure 1:**
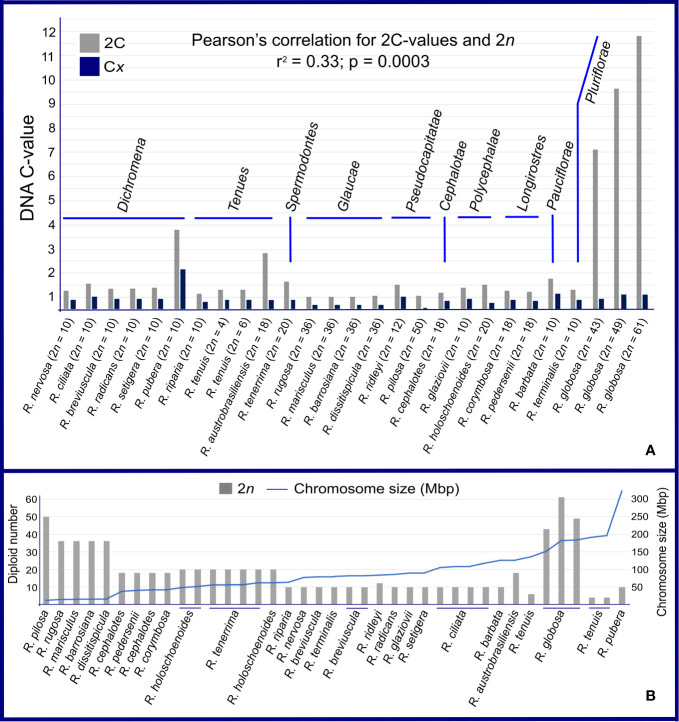
Genome sizes in *Rhynchospora*. **(A)** Comparison between holoploid genome sizes (2C- and 1 Cx-values). DNA content varied throughout the genus, within sections and among taxa with the same chromosome number. Note that section *Glaucae* is the exception, with stable DNA contents and chromosome numbers. **(B)** This graph shows a negative association between 2*n* and average chromosome size in Mbp in almost every sample. The opposite is true for *Rhynchospora globosa*, which presented high chromosome numbers and large chromosomes.

Samples from 39 populations were studied, comprising 24 taxa of *Rhynchospora*. Genome size data and other details are presented in **Figures 1A, B** and **Supplementary data 1**. The 2C-values showed a 22-fold variation between the lowest, 0.51 pg in *R. rugosa* (2*n* = 36), and the highest, 11.32 pg in *R. globosa* (2*n* = 61). Most samples fall within a narrow range of 2C-values (from 0.51 pg to 1.28 pg). Outliers to this parameter are *R. pubera*, *R. tenuis* subsp. *austrobrasiliensis* (referred to as *R. austrobrasiliensis*) and *R. globosa*, all of which have larger holoploid genome sizes. When we evaluated the hypothetical monoploid 1C*x*-value distribution, a difference of about 15× appeared, from 0.06 pg in the polyploid *R. pilosa* (2*n* = 10*x* = 50) to 0.94 pg in the polyploid/dysploid *R. globosa* with 2*n* = 12*x* = 61, and about 27× in relation to the diploid *R. pubera* with 2*n* = 10 (**Figure 1A** and **Supplementary data 1**). Our estimates of nuclear DNA content did not differ much among populations of diploid *R. breviuscula*, *R. nervosa* subsp*. ciliata* (referred to as *R. ciliata*), and tetraploid *R. holoschoenoides* and *R. tenerrima* species. However, different polyploid populations of *R. globosa* had divergent genome sizes.

We compared genome size differences among sections and summarize here the most interesting observations. The sect. *Glaucae* and sect. *Longirostres* have the most constant 2C DNA contents, differing 1.05× and 1.09× in relation to *Dichromena*, *Tenues*, *Pseudocapitatae*, and *Polycephalae*. Four cases drew our attention: i) in sect. *Dichromena*, the diploid *R. pubera* with 2*n* = 10 has 3 times more DNA content than the other diploid species in the section, ii) in sect. *Tenues*, the polyploid *R. austrobrasiliensis*, with 2*n* = 18, exhibited three times more DNA content than *R. tenuis* with 2*n* = 6, iii) in sect. *Pseudocapitatae*, *R. ridleyi* with 2*n* = 12 presented twice the 2C-value as the polyploid *R. pilosa* with 2*n* = 50, and iv) in the polyploid/dysploid *R. globosa* (sect. *Pluriflorae*), the 2C-value variation was superior to the numerical changes involving few chromosomes, i.e. from 43 to 49 (**Figure 1A**). In sect. *Pluriflorae*, a 13.9× difference was observed between *R. terminalis* var. *rosemariana* with 2*n* = 10 and *R. globosa* with 2*n* = 61. *Pearson’s* correlation test between 2C-values and 2*n* chromosome numbers resulted in a low correlation (r^2^ = 0.33; p = 0.0003). Average chromosome sizes varied from 11 Mbp in *R. pilosa* (2*n* = 50) to 320 Mbp in *R. pubera* (2*n* = 10). We found a clear negative correlation between chromosome numbers (2*n*) and average chromosome sizes (**Figure 1B**). Except for *R. globosa*, most species with high chromosome numbers tended to show smaller average chromosome sizes, while species with few chromosomes showed larger average chromosome sizes.

### Karyotype Diversity

**Figure 2:**
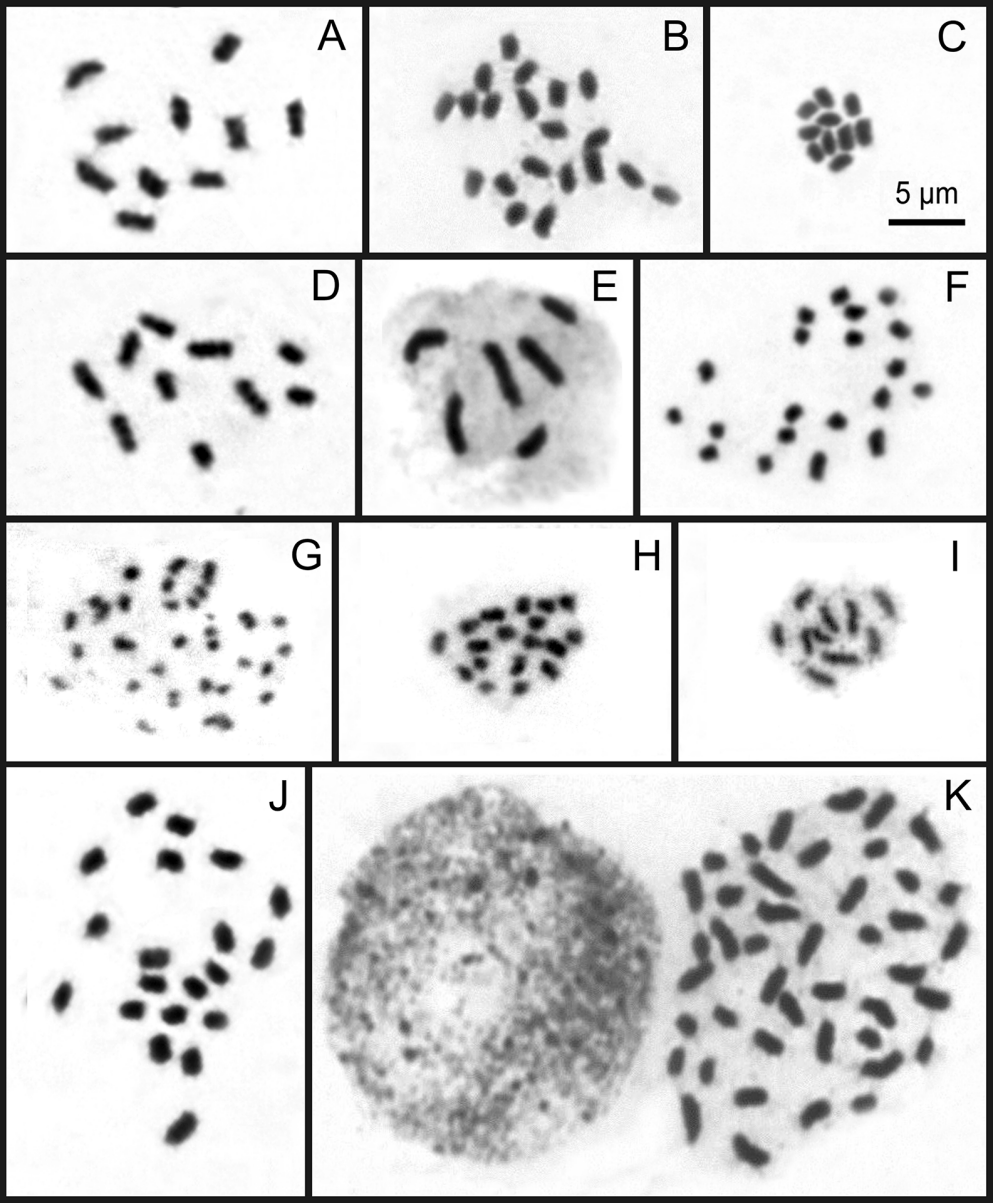
Overview of karyotype diversity in holocentric species of *Rhynchospora*. Mitotic chromosomes of *R. nervosa* with 2*n* = 10 **(A)**, 2*n* = 20 **(B),** and *R. setigera* with 2*n* = 10 **(C)** from sect. *Dichromena.* Note a relative symmetry among of them. Mitotic chromosomes of *R. riparia* with 2*n* = 10 **(D)** and *R. tenuis* with 2*n* = 6 **(E)**, both from sect. *Tenues* and *R. tenerrima* with 2*n* = 20 **(F)** from sect. *Spermodontes.* Note that chromosomes of the polyploid *R. tenerrima* are smaller than those of closer species. The mitotic chromosomes of polyploid *R. dissitispicula* with 2*n* = 36 in **(G)** represents an asymmetrical karyotype with the smaller chromosomes sampled here. Metaphase of *R. pedersenii* (sect. *Longirostres*) with 2*n* = 18 **(H)**. Images in **(I–K)**, from sect. *Pluriflorae*, comprise the most variable karyotypes observed here. Prometaphase in *Rhynhcoposra terminalis* var. *rosemariana* with 2*n* = 10 **(I)** and metaphases in *R. albobracteata* with 2*n* = 20 **(J)** and *R. globosa* with 2*n* = 43 **(K)**, exhibit variability not only in number, but also in the sizes and symmetry of karyotypes. Note the accumulation of chromocenters in *R. globosa* interphase.

Conventional cytogenetic analysis showed a wide variety of karyotypes, with numbers ranging from 2*n* = 4 to 61 and chromosomes differing in size from about 1.3 to 7 μm (**Figure 2**). In this data set, new chromosome counts were reported for *R. albobracteata*, *R. terminalis* var. *rosemariana*, *R. dissitispicula* and *R. pedersenii*, new populations for *R. barbata*, *R. barrosiana*, *R. cephalotes*, *R. corymbosa*, *R. globosa*, *R. holoschoenoides*, *R. marisculus*, *R. nervosa* subsp. *nervosa* (referred to as *R. nervosa*), *R. ciliata*, *R. riparia*, *R. rugosa*, *R. tenuis*, *R. austrobrasiliensis*, and a new chromosome race for *R. tenuis* (2*n* = 6) and cytotypes for *R. globosa* (2*n* = 43, 49, and 61). Chromosome numbers for all the taxa and populations, including some data from literature, are summarized in **Supplementary data 1**. To follow a logical cytotaxonomic order, results are also presented in **Supplementary data 4–7**, according to their phylogenetic relatedness and also considering Kükenthal’s taxonomic classification (1949; 1950a; 1950b; 1951). Within this numerical chromosome variation, numbers derived from *x* = 5 (2*n* = 10, 20, and 30) were the most common. Multiples of *x* = 5 were found in over 63% of the samples, while multiples of *x* = 6 or 9 were registered in a smaller number of accessions (~23% and ~14%, respectively).

Among representatives of sect. *Dichromena*, karyotypes were more symmetrical in relation to the other sections (**Figure 2** and **Supplementary data 4**). Despite this relative chromosome homogeneity, *R. pubera* (**Supplementary data 4**) has chromosomes almost 3× larger than other species, and a twofold size difference among its chromosomes. Contrastingly, all cytotypes in *R. nervosa* (2*n* = 10, 20, and 30) had symmetrical karyotypes (**Supplementary data 4**). In sect. *Tenues* (**Supplementary data 5**), a gradual reduction in chromosome size was observed in all diploid, dysploid, and polyploid species. The chromosome race in *R. tenuis* with 2*n* = 5 stands out for having one chromosome 3 times smaller than the largest chromosome. Section *Spermodontes*, represented here by *R. tenerrima* (**Figure 2F**), showed much smaller chromosomes than those from sect. *Tenues.* Sections *Glaucae*, *Cephalotae*, *Polycephalae*, and *Longirostres* (**Figures 2G, H** and **Supplementary data 6**) exhibited up to twofold differences in chromosome sizes within karyotypes, and also some of the smallest chromosomes in *R. rugosa*, *R. marisculus, R. dissitispicula* and *R. cephalotes* (**Figure 2G** and **Supplementary data 6** **)**. Section *Pluriflorae* exhibited the most significant variation among samples. While the diploid *R. terminalis* and the tetraploid *R. albobracteata* presented symmetrical karyotypes (**Figures 2I, J** **)**, *R. globosa* had strongly asymmetrical karyotypes among its polyploid and dysploid cytotypes (2*n* = 36 to 61) (**Figure 2K** and **Supplementary data 7**).

### C-CMA/DAPI Band Variation

**Figure 3:**
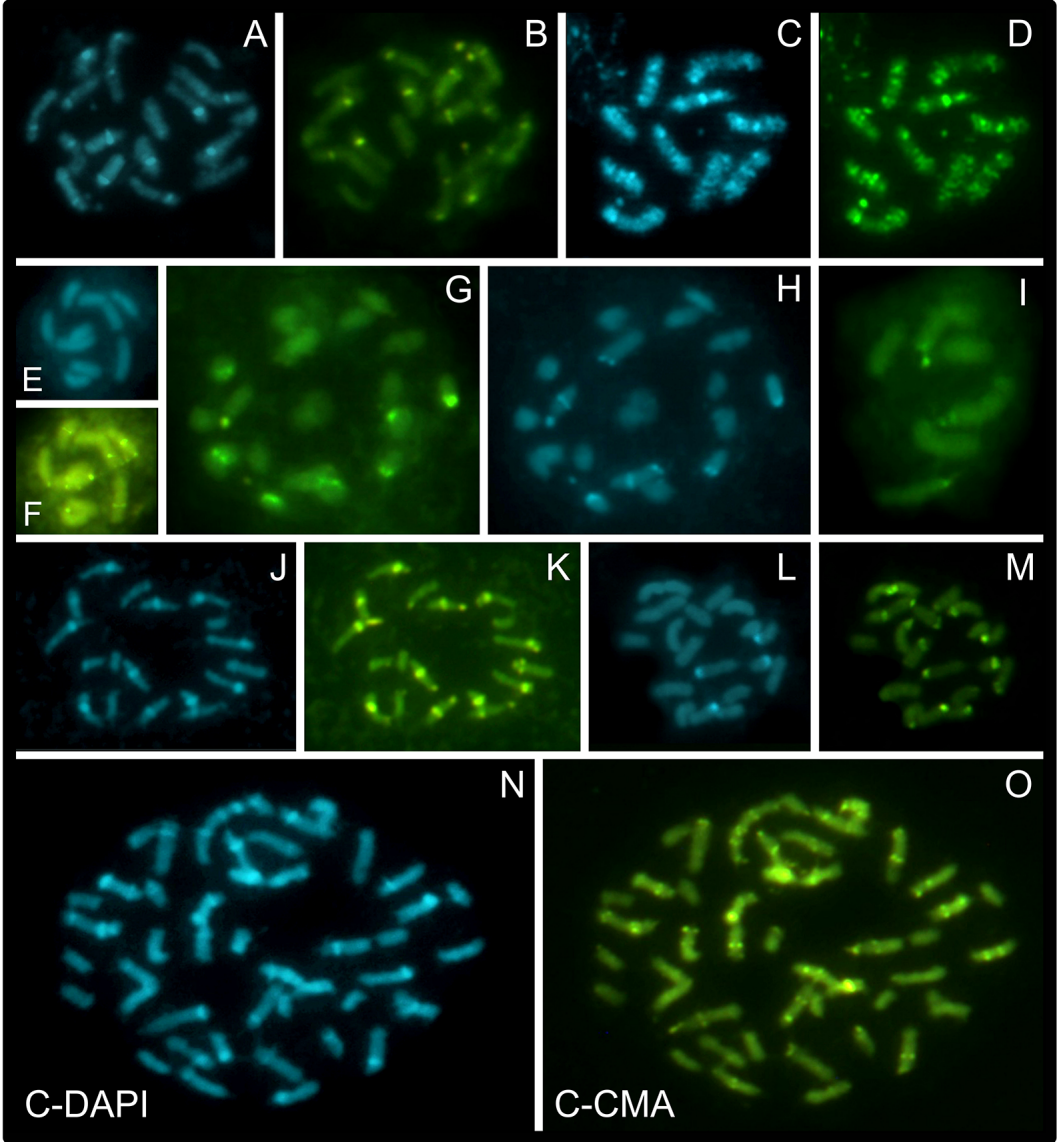
Overview of C-CMA/DAPI banding profiles in holocentric species of *Rhynchospora* containing different chromosome numbers and sizes. Prometaphases of *R. nervosa* from Carrancas with 2*n* = 20 **(A, B)**, *R.* *ciliata* with 2*n* = 10 **(C, D)**, and *R. breviuscula* with 2*n* = 10 **(E, F)**, from sect. *Dichromena*. Note the accumulation of interstitial CMA^+^/DAPI^+^ bands in *R.* *ciliata* and the lack of DAPI^+^ signals in the chromosomes of *R. breviuscula.* Prometaphases in *R. tenerrima* (2*n* = 20; sect. *Spermodontes*) **(G, H)**, and in *R. tenuis* (2*n* = 6; sect. *Tenues*) **(I)**. While *R. tenerrima* showed both CMA^+^ and DAPI^+^ bands, a closely related species, *R. tenuis*, only has CMA^+^ signals. Prometaphase of *R. corymbosa* with large interstitial DAPI^+^/CMA^+^ blocks in several chromosomes, and some smaller terminal DAPI^0^/CMA^+^ ones **(J, K)**. Metaphase of *R. albobracteata*, with many terminal CMA^+^ and fewer terminal DAPI^+^ bands **(L, M)**, and prometaphase of *R. globosa* with 2*n* = 43, from Jaguariaíva **(N, O)**, both from sect. *Pluriflorae*. Note that band distribution in *R. globosa* is more diverse that in *R. albobracteata*.

**Figure 4:**
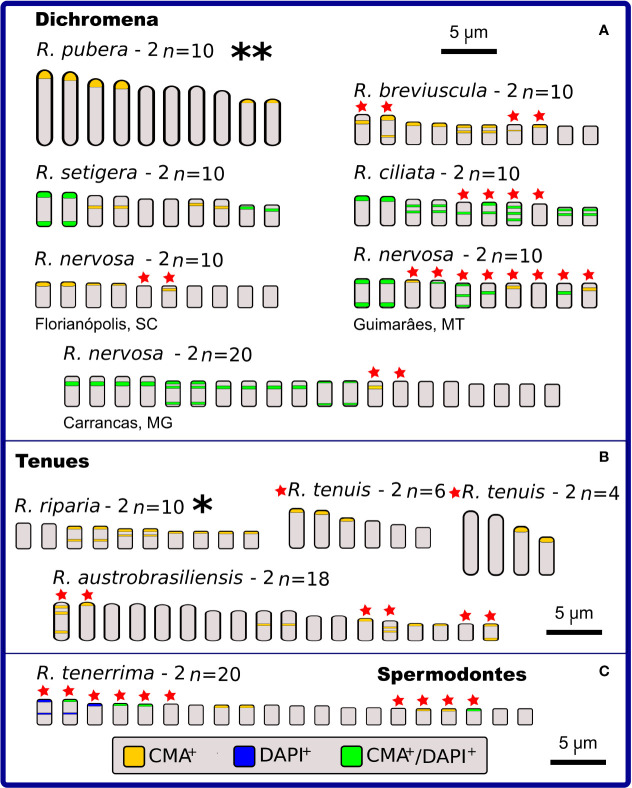
Idiograms representing the physical mapping of C-CMA/DAPI banding in karyotypes of 12 species of *Rhynchospora*, including two data obtained from literature (Vanzela and Guerra, 2000), which are highlighted with * and **. Species are grouped in sections according to Kükenthal’s classification. In sect. *Dichromena* **(A)**, five cytotypes exhibited polymorphisms in the banding location (indicated by red stars). Observe the large C-CMA/DAPI banding diversity among populations of *R. nervosa*. Only *R. pubera* and *R. setigera* showed regular distribution of bands. From the other two closest sections (*Tenues* and *Spermodontes*, **(B, C)**, respectively), only *R. riparia* exhibited a regular band distribution. *Rhynchospora tenuis* (2*n* = 4 and 2*n* = 6) presents karyotypes completely involved in chromosome fusion (symploidy) and fission (agmatoploidy) and the other two, *R. austrobasiliensis* and *R. tenerrima*, showed part of their chromosomes with heteromorphisms.

**Figure 5:**
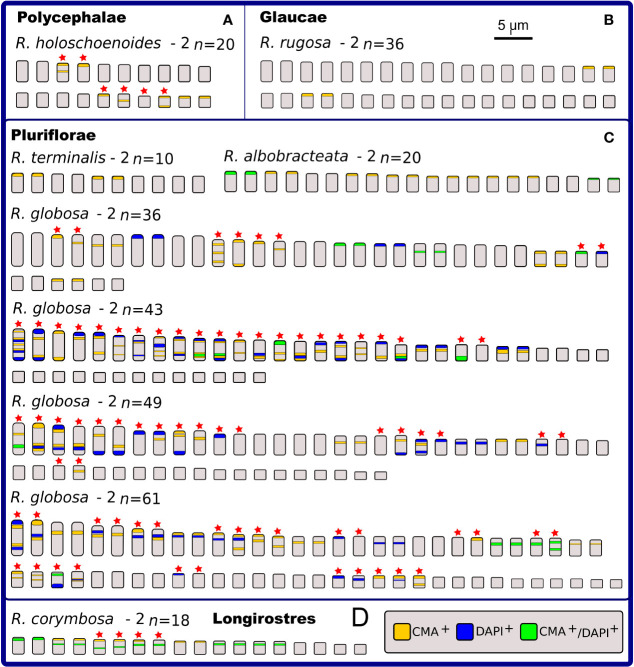
Idiograms representing the physical mapping of C-CMA/DAPI banding in karyotypes of nine species of *Rhynchospora* from sections *Polycephalae* **(A),** *Glaucae* **(B),** *Pluriflorae* **(C),** and *Longirostres* **(D),** according to Kükenthal’s classification. Except for *R. rugosa* **(B),** *R. terminalis* and *R. albobracteata* **(C),** the remaining species exhibit polymorphisms in the banding location (indicated by red stars). The most striking situation occurs in the populations of *R. globosa*, which vary in chromosomal number (polyploidy and dysploidy) and in the banding profiles, i.e. number of terminal and interstitial bands, in addition to large differences in the occurrence and number of CMA^+^, DAPI^+^, and CMA^+^/DAPI^+^.

Chromosome banding was performed to check for possible differences and similarities among karyotypes, in addition to the features already observed by conventional cytogenetics. As criteria, the occurrence, number and position of CMA^+^, DAPI^+^, and CMA^+^/DAPI^+^ bands were evaluated. Fifteen taxa were analyzed, and a high diversity of band profiles was observed (**Figures 3**–**5**). Variation included number as well as location (terminal, subterminal, and/or interstitial), and this was evident when we compared different populations of *R. nervosa* and *R. globosa*. In sect. *Dichromena*, interstitial or terminal/subterminal CMA^+^/DAPI^+^ or CMA^+^/DAPI^0^ bands were common to all species, and the accumulation of interstitial bands in *R. ciliata* was visible. CMA^+^/DAPI^+^ was observed only in *R. setigera*, *R. ciliata* (both with 2*n* = 10), and a population of *R. nervosa* with 2*n* = 10 and another with 2*n* = 20 (**Figures 3A–F** and **Supplementary data 8**). *Rhynchospora pubera*, the species with the largest chromosomes and genome size in sect. *Dichromena*, accumulated fewer bands than other species with smaller genomes (**Figure 4A**).

*Rhynchospora tenerrima* (sect. *Spermodontes*), which is the closest species to sect. *Tenues*, exhibited both CMA^+^ and an evident interstitial DAPI^+^ band (**Figures 3G, H** and **4C**). In comparison to species from sect. *Tenues*, terminal CMA^+^ bands prevailed, except for *R. austrobrasiliensis* which has insterstitial bands (**Figures 3G–I**, **4B** and **Supplementary data 9A–F**). Samples from sect. *Glaucae*, *Polycephalae* and *Longirostres* (**Figures 3** and **5** and **Supplementary data 10**) showed a predominance of terminal CMA^+^ bands, with few interstitial CMA^+^ bands in *R. holoschoenoides* (**Supplementary data 10E, F**). It is important to highlight that *R. corymbosa* from sect. *Longirostres* accumulated several terminal and interstitial CMA^+^/DAPI^+^ bands (**Supplementary data 10C, D** and **Figure 5D**). Species from sect. *Pluriflorae* presented variable banding profiles, specially samples of *R. globosa* (**Figures 3L–O**, **5C**, and **Supplementary data 11** **)**. The absence of DAPI^+^ bands in *R. terminalis*, the accumulation of CMA^+^ and CMA^+^/DAPI^+^ in *R. albobracteata* (**Supplementary data 10G–J**), and a highly variable banding distribution in *R. globosa* (see **Figure 5C** and **Supplementary data 11**) were evident.

In relation to intraspecific diversity and polymorphisms in C-CMA/DAPI bands, we compared diploid/polyploid samples of *R. nervosa* (**Figure 4A**) with cytotypes of *R. globosa* (**Figure 5C**), which is a complex of polyploid/dysploid taxa. The amount and position of CMA^+^ and CMA^+^/DAPI^+^ bands varied among diploid samples of *R. nervosa*. Besides, the band profile observed in the polyploid did not represent the exact duplication of band profiles of diploids. Polymorphisms were observed in all three cases (**Figure 4A**). Populations of *R. globosa* were the most variable regarding band profiles, which often made chromosome pairing impossible. We can highlight the absence of bands on the two largest chromosomes and the presence of bands on the smallest chromosomes in the population with 2*n* = 36, and the inverse situation in populations with 2*n* = 43, 49, and 61, where the smallest ones have no bands and the largest ones accumulate more heterochromatic bands (**Figure 5C**). Polymorphisms in the occurrence and location of CMA and/or DAPI bands were detected in nine of the 15 species compared (indicated as red stars in **Figures 4** and **5**).

### Phylogenetic Relationships and Ancestral Chromosome Number (ACN) Reconstruction

**Figure 6:**
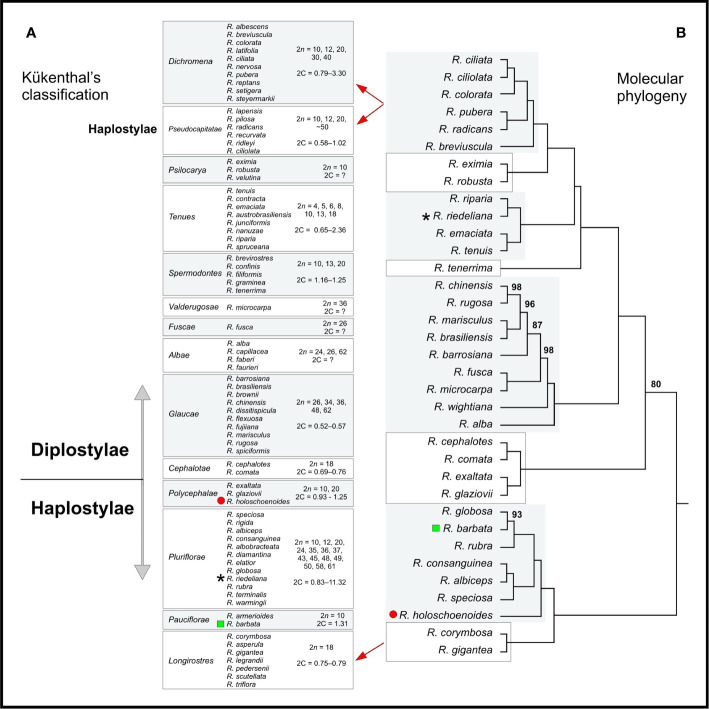
**(A)** Kükenthal’s classification is contrasted with **(B)** the maximum likelihood phylogenetic inference for Rhynchospora based on 54 chloroplast coding sequences (CDS), Length: 51,383 bases. The red circle, the black star and the green square indicate species whose phylogenetic position differed from Kükenthal’s classification. Numbers above the branches indicate bootstrap values. Branches without numbers have bootstrap values of 100. The range of 2*n* values for each clade is shown from highest to lowest. Taxa and 2*n* values contain more taxa than are shown in the phylogeny—the clades are considered to be representative for the taxonomic sections.

Species relationships were obtained for 37 taxa from a plastid concatenated alignment >50 Kb in length comprising >1,300 sequences. Despite the limited amount of species, a large number (54) of chloroplast coding loci were used, and the tree showed high support (>95 bootstrap) for most clades. All *Rhynchospora* taxa for which chromosome numbers and genome sizes are available were organized according to Kükenthal’s classification and are presented beside the phylogenetic tree obtained from chloroplast sequences (**Figure 6**). Comparing **Figures 6A, B**, the majority of taxa in Kükenthal’s sections were assigned to the same phylogenetic clade. In general, the phylogenetic analysis was in agreement with the previous taxonomic proposition regarding pars Diplostylae and Haplostylae, except for sect. *Pseudocapitatae* (Haplostylae) that grouped with sections belonging to pars Diplostylae. *Rhynchospora holoschoenoides*, *R. riedeliana*, and *R. barbata* were grouped with different taxa than Kükenthal had proposed. Also, sect. *Dichromena* and *Pseudocapitatae* were assigned to the same phylogenetic clade. Within every section/clade, taxa share the same basic chromosome number, however, their distribution along the phylogenetic tree does not seem to follow a particular order, neither does the occurrence of dysploidy or polyploidy, which are common in almost every clade, i.e., numerical and structural rearrangements appeared in all the clades.

**Figure 7:**
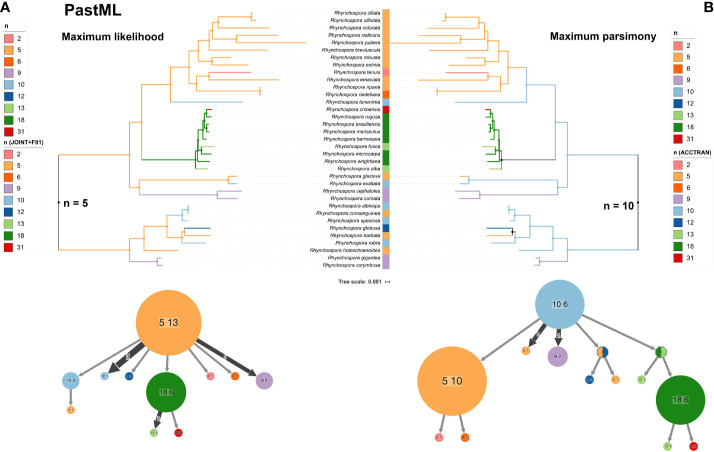
Ancestral chromosome number reconstruction with PastML along the same phylogeny shown in with the two equally best prediction methods. **(A)** Left panel shows the maximum likelihood JOINT+F81 and **(B)** the right panel shows the maximum parsimony accelerated transformation prediction methods, respectively. Bottom graphs show the PastML compressed nodes view for each ancestral character reconstruction method.

To gain insights into major drivers of chromosome evolution in *Rhynchospora* we analyzed the frequency of chromosome number change events and performed an ancestral chromosome number reconstruction based on the phylogenetic tree with PastML (**Figure 7**) and ChromEvol (**Supplementary data 12**). Based on the ancestral character reconstruction with PastML (Ishikawa et al., 2019) karyotypes with *n* = 5 or 10 were the most likely ancestral complement, based on maximum likelihood (ML) and maximum parsimony (MP), respectively (**Figure 7**). The ChromEvol model reported an ACN of *n* = 5 and variations were mostly attributed to polyploidization (1.39), chromosome fusion (1.13) and less frequently to fission (0.76) (**Supplementary data 12** shows much more support for *n* = 5), according to the optimal model selected by means of the Akaike information Criterion (AIC) (Akaike, 1974). N = 5 was found as the most likely ACN in most clades with both ChromEvol and PastML ML JOINT+F81 models, except the species rich clade corresponding to sections *Glaucae*-*Albae*-*Fuscae*-*Valderugosae*, for which the ACN was *n* = 9 and *n* = 18 for ChromEvol and ML JOINT+F8, respectively. Results obtained with PastML MP showed *n* = 10 as the most likely ACN in most clades, again only the clade *Glaucae*-*Albae*-*Fuscae*-*Valderugosae* did not converge to *n* = 10.

## Discussion

### Karyotype Differentiation Versus DNA Content Variation

Genomes size analyses allow us to understand the DNA gain and loss influence among related species, and can explain some aspects of evolutionary differentiation among taxa (Bennett and Leitch, 2005; Leitch et al., 2010). The study of DNA content in holocentrics can lead to interesting results, as genome sizes can be maintained, or not, in clades with regular or rearranged karyotypes (Roalson, 2008), such as observed in *R. tenuis* with 2*n* = 4 (0.80 pg) and 2*n* = 6 (0.83 pg). DNA amount varied by approximately 22× in 2C-values and 27× in hypothetical 1C*x*-values. However, a comparison between three sections showed that the predominantly hexaploid sect. *Glaucae* (2*n* = 36 and 0.51–0.56 pg) proved to be very stable in relation to sect. *Dichromena* (diploid and polyploid) (2*n* = 10, 20 or 30, and 0.78–3.3 pg) and sect. *Tenues* (diploid, dysploid, and polyploid). The latter two sections have two species (*R. pubera* and *R. austrobrasiliensis*, respectively) that stood out for their substantial genomic DNA accumulation. The great diversity in genome sizes becomes clearer when the putative diploid species *R. terminalis* and *R. ridleyi*, and the assumed polyploids *R. pilosa* and *R. globosa*, are contrasted. A noteworthy case was the ~17% contrast in genome size between assumed tetraploid populations of *R. holoschoenoides*, with 1.25 pg (Ribeiro et al., 2018) and 1.06–1.10 pg. This may be an indication of extensive genome differentiation between populations sampled over 3,000 kilometers apart. Fluctuations in DNA content in Cyperaceae have already been linked to the activity of the transposable elements (Bureš and Zedek, 2014; Souza et al., 2018). In addition, there is evidence of differential accumulation of 35S rDNA sites and heterochromatic bands in *Rhynchospora* and *Eleocharis* (Vanzela et al., 1998; Vanzela and Guerra, 2000; Da Silva et al., 2008; Da Silva et al., 2010). This allows us to suggest that variations in the repetitive DNA fraction can contribute to karyotype differentiation in *Rhynchospora*.

Regarding karyotype organization, numerical diversification in sect. *Tenues* seems to have happened *via* a set of events, starting from 2*n* = 10 (*n* = 5), reducing to 2*n* = 4 (*n* = 2; descending dysploidy), and achiasmatic meiosis in *R. tenuis* (Vanzela et al., 2003; Cabral et al., 2014), followed by ascending dysploidy to 2*n* = 5, 6, and a possible polyploid with 2*n* = 8, also in *R. tenuis*. Besides, potential polyploidy was responsible for 2*n* = 18 found in two other species in the section (see Vanzela et al., 1996; Vanzela et al., 2000; Arguelho et al., 2012; Michelan et al., 2012). Except for the potential polyploid cases, we do not see such a sharp contrast in DNA content between diploid and dysploid species of this section. Intraspecific analysis of different populations of *R. globosa* with different chromosome numbers (2*n* = 43, 49, and 61) showed that chromosome number increases are not associated with proportionate genome size increases. Unlike what happened in sect. *Tenues*, polyploidy, dysploidy, and variations in the repetitive fraction of DNA may be acting together in *R. globosa* 2C DNA content variation, and this can be seen in the hypothetical 1C*x-*values. When we compare the DNA amount variation (22–27×) with other Cyperaceae, *Rhynchospora* spp. are more variable than *Carex*, which exhibits a 7× difference among species (Lipnerová et al., 2013), and closer to what happens in *Eleocharis* which exhibited a 21.43× variation (Zedek et al., 2010; Souza et al., 2018). Dysploidy is common in *Rhynchospora* as well as in other Cyperaceae where there is a predisposition for cytotypes and chromosome races in its various clades, including odd numbers due to chromosome fission and fusion, associated or not with polyploidization (Mola and Papeschi, 2006; Roalson, 2008). This could explain the occurrence of different basic numbers (*x* = 5, 6, and 9) in *Rhynchospora* (Luceño et al., 1998a; Vanzela et al., 2000; Ribeiro et al., 2018), as well as in other genera of this family (see Roalson, 2008).

### DNA Content Diversity Versus CMA/DAPI Band Gain and Loss

Results focusing on the C-CMA/DAPI bands allowed us to access the dynamics of holocentric genomes from another point of view. Our work with *Rhynchospora* provides one more line of evidence that heterochromatic band accumulation and elimination have an important role in genome differentiation and DNA content fluctuations in plants generally, and specifically in Cyperaceae (Kellogg and Bennetzen, 2004; Grover and Wendel, 2010; Bureš and Zedek, 2014). The comparison among C-CMA/DAPI band profiles suggests an association between band accumulation and increase of DNA 2C-values in some species, but not in others. This is evident in *R. breviuscula*, *R. ciliata*, and *R. pubera* (see also Vanzela and Guerra, 2000; Ribeiro et al., 2018), since *R. ciliata* exhibits many C-CMA/DAPI bands scattered throughout its chromosomes and unlike *R. pubera* which has threefold larger genome and few bands. Although these suggest that accumulation of repetitive sequences is not detectable by banding techniques in *R. pubera*, the data obtained here do not provide much insight about the nature of these sequences. It is known that not every family of satellite DNA is detected by banding. A good example of a region unlikely to be detected is the centromeric satDNA Tyba that specifically associates with CENH3 (the centromeric histone H3 variant) within the groove along the holocentric chromatids of *R. pubera* (Marques et al., 2015). Only non-centromeric satellite sequences were identified as heterochromatic blocks, as the ones found in *R. globosa* and *R. ciliata* (Ribeiro et al., 2017). When we compared karyotypes in the same taxonomic section, but with large genome size variation (*R. tenuis* and *R. austrobrasiliensis*), or polyploid species from distant sections (*R. nervosa*, *R. tenerrima*, *R. holoschoenoides* and *R. albobracteata*), it is possible to note that there is not always CMA/DAPI band amplification when chromosome number increase.

Polymorphisms were found after C-CMA/DAPI banding in nine out of the 15 species analyzed. Indeed, this peculiarity was observed before in *R. ciliata* and *R. tenuis* (Ribeiro et al., 2017). In several cases, homologous chromosome pairs could not be identified as they did not present identical banding patterns, even when there is normal bivalent formation at meiosis, such as *R. ciliata* (Luceño et al., 1998a), *R. breviuscula*, and *R. nervosa* (Arguelho et al., 2012). These observations suggest that a reduced level of meiotic recombination or unequal recombination could favor accumulation of heterozygosity between homologs. In addition, the apparent regular axis formation observed in the meiosis of some *Rhynchospora* species (Cabral et al., 2014), could be enough to enable regular pairing within diploid and polyploid species, even in those with differences in sequence collinearity. However, this does not seem to apply to our samples of *R. globosa* that accumulated many band polymorphisms, associated with intense numerical rearrangements, and in which there is evidence of irregular meiosis (Luceño et al., 1998a; Arguelho et al., 2012).

### Insights on Genome Evolution of Holocentric Species of *Rhynchospora*

The phylogenetic analysis using a large fraction of chloroplast genomes, combined with chromosome and genome size data, showed a wide diversity among and within sections of *Rhynchospora*, similar to the findings of Ribeiro et al. (2018). Species were placed in equivalent clades, except for *R. cephalotes* and *R. exaltata*, which were grouped together in a single clade. The ChromEvol analysis indicated polyploidization as the major driver of genome evolution in *Rhynchospora*, followed by dysploidy. Ancestral chromosome number reconstruction gave either *x* = 5 or *x* = 10 as potential ACN in contrast to the primary number *x* = 5, reported by classic cytogenetic studies (Luceño et al., 1998a; Vanzela et al., 2000; Arguelho et al., 2012; Michelan et al., 2012). If *x* = 5 is indeed the ACN for *Rhynchospora* (as suggested by the Bayesian ChromEvol method), then polyploidy could be more relevant than fission/fusion events, although we could not discard other mechanisms. If *x* = 10 is the ACN, dysploidy would be the most active mechanism. However, the low number of populations analyzed until now could actually “mask” the intraspecific variability and the importance of dysploidy in *Rhynchospora* evolutionary history. Some chromosome pairing reports in species with 2*n* = 18 and 2*n* = 20 always showed bivalents (Luceño et al., 1998a; Luceño et al., 1998b; Vanzela et al., 2000). Because 10 appeared as a secondary ACN in our maximum parsimony analysis, this could suggest a diploid meiotic behavior or then point toward a possible paleopolyploidy. In this case, we cannot exclude *n* = 10 as a candidate for ACN in *Rhynchospora*.

Chromosome numbers and genome sizes were negatively correlated overall, though there was a lack of consistence between closely related species. This could be explained by changes associated with differential repetitive DNA accumulation associated with numerical rearrangements. Although transposable elements were not evaluated in this study, changes in genome size mediated by proliferation of retrotransposons were reported as driving genomic changes in sedges (Zedek et al., 2010; Lipnerová et al., 2013; Souza et al., 2018; Johnen et al., 2020), and this could be the focus of future studies in *Rhynchospora*. This idea is supported by the fact that other non-heterochromatic repetitive sequences played a role in differentiating some genomes, such as *R. ciliata* and *R. pubera* (Marques et al., 2015; Ribeiro et al., 2017), besides it appears that C-CMA/DAPI heterochromatin changes independently of phylogenetic relationships. Perhaps the best indication of this diversity are differences between the large genome of *R. pubera* with 2*n* = 10 and 320 Mbp of average chromosome size and species of section *Glaucae*, with 2*n* = 36 and 14 Mbp of chromosome size and *R. pilosa* with 2*n* = 50 and 11 Mbp of chromosome size. An exception to this rule is *R. globosa*, in which many large chromosomes are found, likely representing a true polyploid.

Indeed, such scenarios also seem to happen in other holocentric organisms, and it could be, at least in part, explained by the holokinetic drive model (Bureš and Zedek, 2014). The proposed model works similarly to the centromere drive model of Henikoff et al. (2001), but instead of facilitating evolution of centromere size (number and symmetry), it would facilitate changes in chromosome size and number. Under the holokinetic drive model there are two competing tendencies, 1) fission and loss of repetitive elements and 2) fusion and accumulation of repetitive elements. Similar to the centromeric drive, the holokinetic drive would also depend on i) meiotic asymmetry and ii) the asymmetry of the egg and polar body poles (Bureš and Zedek, 2014). *Rhynchospora* and other Cyperaceae show meiotic asymmetry in both female and male meiosis (Rocha et al., 2016), which could potentially amplify the effectiveness of holokinetic drive in the family. Since *Rhynchospora* genomes are composed of repeat-rich holocentromeres, each duplication/unequal crossing-over could potentially generate chromosomes that accumulate (or lose) more centromere units, facilitated by asymmetric meiosis. In the future, comparative genomic analysis will hopefully unveil the mechanisms for genome evolution in holocentric plants.

## Data Availability Statement

All datasets generated for this study are included in the article/supplementary material.

## Author Contributions

AV: directed researches. PB and MG: performed conventional cytogenetic, C-CMA/DAPI, and flow cytometry analyses. PB, CB, and AM performed bioinformatic and phylogenetic analyses. AV and MS: helped with conventional, C-CMA/DAPI, and idiogram analyses. AV, PB, AM, CB, and MG: wrote the manuscript.

## Conflict of Interest

The authors declare that the research was conducted in the absence of any commercial or financial relationships that could be construed as a potential conflict of interest.

The reviewer TJ declared a past co-authorship with one of the authors, AM.
