# Supplementary material for: Holocentric Karyotype Evolution in Rhynchospora Is Marked by Intense Numerical, Structural, and Genome Size Changes
Source: Front Plant Sci. 2020 Sep 10;11:536507. doi: 10.3389/fpls.2020.536507 (PMC7533669; doi:10.3389/fpls.2020.536507)
Supplement: Supplementary file 1 [file DataSheet_1.pdf]

SUPPLEMENTARY MATERIAL: Burchardt et al. 2020. Holocentric karyotype evolution in *Rhynchospora* (Cyperaceae) is marked by intense numerical and structural changes, and in genome sizes.

Supplemental data 1. Genome and karyotype information of *Rhynchospora* species organized in sections according Kukenthal's classification

Sections	Species/Samples	2*n*	*n*	*x*	2C ± SD (pg)	1C (Mbp)	Locality	References/new vouchers
Dichromena	*R. wightiana* (Nees) Steudel	–	10	5	–	–	–	Rath and Patnaik (1978)
	R. albescens (Miqu.) Kük.	–	*c.* 10	–	–	–	–	Thomas (1984)
	R. breviuscula H.Pfeiff.	10	5	5	–	–	–	Luceño *et al.* (1998)
		10	5	5	–	–	–	Arguelho *et al*. (2012)
		10	5	5	0.83 ± 0.02	405.87	Guapiara, SP	FUEL056045
		10	5	5	0.80 ± 0.02	391.20	Eldorado, SP	FUEL056044
		10	5	5	0.83 ± 0.03	405.87	Iporanga, SP	FUEL055362
	R. colorata (L.) Hitchc.	–	5	5	–	–	–	Thomas (1984)
	R. latifolia (Baldw.) Thomas	–	5	5	–	–	–	Thomas (1984)
	R. nervosa subsp. *nervosa* (Vahl) Böckeler	10	5	5	–	–	–	Arguelho *et al*. (2012)
		10	5	5	–	–	Chapada dos Guimarães, MT	FUEL056127
		10	5	5	0.78 ± 0.01	381.42	Florianópolis, SC	FUEL056046
		20	10	5	–	–	–	Luceño *et al.* (1998)
		20	10	5	–	–	–	Arguelho *et al*. (2012)

Table continuation....							
	20	10	5	–	–	Carrancas, MG	FUEL059059
	30	15	5	–	–	–	Luceño *et al.* (1998)
	30	15	5	–	–	–	Arguelho *et al*. (2012)
	–	20	5	–	–	–	Shibata (1962)
R. nervosa subsp. *ciliata* T.Koyama	10	5	5	1.12	–	–	Ribeiro *et al*. (2017)
	10	5	5	–	–	–	Luceño *et al.* (1998)
	10	5	5	–	–	–	Arguelho *et al*. (2012)
	10	5	5	1.11 ± 0.03	542.79	UFPB, Areia, PB	FUEL056048
	10	5	5	1.10 ± 0.02	537.90	Areia, PB	FUEL056047
	10	5	5	1.07 ± 0.03	523.23	Recife. PE	FUEL055372
R. pubera (Vahl) Boeckeler	10	5	5	3.3	1,613.70	–	Marques *et al.* (2015)
	10	5	5	–	–	Recife, PE	FUEL055374
	10	5	5	–	–	–	Luceño *et al.* (1998)
	10	5	5	–	–	–	Arguelho *et al*. (2012)
	12	dysploid	6	–	–	–	Arguelho *et al*. (2012)
R. reptans (Rich.) Böckeler	–	*c.* 20	5	–	–	–	Thomas (1984)
R. setigera (Kunth.) Boeck.	10	5	5	–	–	–	Luceño *et al.* (1998)

Table continuation....

		10	5	5	0.92 ± 0.01	449.88	Jaguariaíva, PR	FUEL056065
		20	10	5	–	–	–	Luceño *et al.* (1998)
	R. steyermarkii Koyama	–	*c.* 10	5	–	–	–	Thomas (1984)
Pseudocapitatae	*R. ciliolata* Boeck.	10	5	5	–	–	–	Vanzela *et al.* (2000)
	R. lapensis C.B. Clarke	20	10	5	–	–	–	Vanzela *et al.* (2000)
	R. pilosa Boeckeler	*c.* 50	25	5	–	–	–	Vanzela *et al.* (2000)
		–	–	–	0.57 ± 0.03	278.73	Carrancas, MG	FUEL055368
	R. radicans (Schltdl. and Cham.) H. Pfeiff.	10	5	5	0.87 ± 0.01	425.43	–	Ribeiro *et al.* (2018)
	R. recurvata (Nees) Steudel	10	5	5	–	–	–	Vanzela *et al.* (2000)
	R. ridleyi C.B. Clarke	12	6	6	–	–	–	Vanzela *et al.* (2000)
		12	6	6	1.02 ± 0.01	498.78	–	Ribeiro *et al.* (2018)
Psilocarya	*R. eximia* (Nees) Boeck.	10	5	5	–	–	–	Vanzela *et al.* (2000)
	R. robusta (Kunth.) Boeck	10	5	5	–	–	–	Vanzela *et al.* (2000)
	R. velutina (Kunth.) Boeck.	10	5	5	–	–	–	Vanzela *et al.* (2000)
		10	5	5	–	–	–	Arguelho *et al*. (2012)
Tenues	*R. contracta* (Nees) Raynal	18	9	9	–	–	–	Vanzela *et al.* (2000)
	R. emaciata (Nees) Boeck.	10	9	5	–	–	–	Vanzela *et al.* (2000)

Table continuation....							
R. junciformis	18	9	9	–	–	–	Arguelho *et al*. (2012)
R. nanuzae Luceño and Rocha	10	5	5	–	–	–	Vanzela *et al*. (2000)
R. riparia (Nees) Boeckeler	10	5	5	–	–	–	Vanzela *et al*. (2000)
	10	5	5	0.64 ± 0.01	312.96	Parque Guariba, PB	FUEL056070
R. spruceana C.B.Clarke	13	dysploid	5	–	–	–	Gadela and Kliphuis (1963)
R. tenuis Link	4	dysploid	5	0,78	381.42	–	Feitoza *et al*. (unpublished data)
	4	dysploid	5	–	–	–	Vanzela *et al*. (2000)
	4	dysploid	5	–	–	–	Arguelho *et al*. (2012)
	4	dysploid	5	0.80 ± 0.02	391.20	Itapoá, PR	FUEL056042
	5	dysploid	5	–	–	–	Arguelho *et al*. (2012)
	6	dysploid	5	0.83 ± 0.04	405.87	Carrancas, MG	FUEL056043
	8	dysploid	5	–	–	–	Vanzela *et al*. (1996)
	10	5	5	–	–	–	Gadela and Kliphuis (1964)
R. tenuis subsp. *austrobrasiliensis* T.Koyama	18	dysploid	5	–	–	–	Vanzela *et al*. (2000)
	18	dysploid	5	–	–	–	Arguelho *et al*. (2012)
	18	dysploid	5	2.32 ± 0.03	1134.48	Antonina, PR	FUEL056071

Table continuation....								
Spermodontes	*R. brevirostres* Griseb.	10	5	5	–	–	–	Vanzela *et al.* (2000)
		10	5	5	–	–	–	Vanzela *et al.* (2000)
	R. confinis (Nees) C.B. Clarke	20	10	5	–	–	–	Vanzela *et al.* (2000)
	R. filiformis Vahl	10	5	5	–	–	–	Vanzela *et al.* (2000)
	R. graminea Uitt.	13	dysploid	5	–	–	–	Gadela and Kliphuis (1964)
	R. tenerrima Nees ex Spreng.	20	10	5	–	–	–	Vanzela *et al.* (2000)
		20	10	5	–	–	–	Arguelho *et al*. (2012)
		20	10	5	1.14 ± 0.05	557.46	Antonina, PR	FUEL056041
		–	–	5	1.14 ± 0.03	557.46	Areia, PB	FUEL056069
		20	10	5	1.13 ± 0.03	552.57	Tupã, SP	FUEL056040
		20	10	5	1.25 ± 0.01	611.25	–	Ribeiro *et al.* (2018)
Valderugosae	*R. microcarpa* Baldw. ex A. Gray	36	18	6	–	–	–	Vanzela *et al.* (2000)
Fuscae	*R. fusca* (L.) Aiton f.	26	13	6	–	–	–	Löve and Löve (1982)
Albae	*R. alba* (L.) Vahl	26	13	6	–	–	–	Gadela and Kliphuis (1963)
		26	13	6	–	–	–	Dietrich (1972)
		26	13	6	–	–	–	Vachova (1976)
		26	13	6	–	–	–	Pogan *et al*. (1980)

Table continuation....								
		26	13	6	–	–	–	Löve and Löve (1981)
		26	13	6	–	–	–	Löve and Löve (1982)
		26	13	6	–	–	–	Hoshino (1987*b*)
		–	13	6	–	–	–	Taylor and Mulligan (1968)
		–	13	6	–	–	–	Pojar (1973)
	R. capillacea Torrey	26	13	6	–	–	–	Löve and Löve (1981)
	R. faberi C.B. Clarke	24	12	6	–	–	–	Hoshino (1987*b*)
	R. faurieri Franch.	62	31	–	–	–	–	Hoshino (1987*b*)
Glaucae	*R. barrosiana* Guagl.	36	18	6	–	–	–	Vanzela *et al.* (2000)
		36	18	6	0.54 ± 0.02	264.06	Tibagi, PR	FUEL056061
	R. brasiliensis Boeck.	*c.* 36	–	6	–	–	–	Vanzela *et al.* (2000)
	R. brownii Roem. and Sch.	34	17	6	–	–	–	Hoshino (1987*b*)
	R. brownii subsp. *americana* Guaglianone	36	18	6	–	–	–	Vanzela *et al.* (2000)
	R. chinensis Böckeler	62	31	6	–	–	–	Hoshino (1987*b*)
	R. dissitispicula T.Koyama	36	18	6	0.56 ± 0.02	273.84	Carrancas, MG	FUEL056050
	R. flexuosa C.B. Clarke	36	18	6	–	–	–	Vanzela *et al.* (2000)
	R. fujiiana Mak.	26	13	6	–	–	–	Hoshino (1987*b*)

Table continuation....								
	R. marisculus Lindl. and Nees	30	15	6	–	–	–	Dopchiz et al. (2000)
		36	18	6	–	–	–	Vanzela *et al.* (2000)
		36	18	6	–	–	–	Arguelho *et al*. (2012)
		36	18	6	0.53 ± 0.01	259.17	Parque Guariba, PB	FUEL056051
	R. rugosa (Vahl) Gale	36	18	6	–	–	–	Vanzela *et al.* (2000)
		36	18	6	0.51 ± 0.03	249.39	Carrancas, MG	FUEL056067
	R. spiciformis Hillebr.	48	24	6	–	–	–	Skottsberg (1955)
Cephalotae	*R. cephalotes* (L.) Vahl	17	dysploid	9	–	–	–	Luceño et al. (1998b)
		18	9	9	–	–	–	Luceño et al. (1998b)
		18	9	9	–	–	–	Vanzela *et al.* (2000)
		18	9	9	0.68 ± 0.02	332.52	Areia, PB	FUEL056039
		19	dysploid	9	–	–	–	Luceño et al. (1998b)
		18	9	9	0.76 ± 0.01	371.64	–	Ribeiro *et al.* (2018)
	R. comata (L.) Roemer and Schultes	18	9	9	–	–	–	Vanzela *et al.* (2000)
Polycephalae	*R. exaltata* Kunth	20	10	5	–	–	–	Luceño *et al.* (1998)
		20	10	5	–	–	–	Luceño *et al.* (1998)
	R. glaziovii Boeckeler	10	5	5	–	–	–	Luceño *et al.* (1998)

Table continuation....								
		–	–	–	0.91 ± 0.01	444.99	Campina Grande do Sul, PR	FUEL056036
	R. holoschoenoides (Rich.) Herter	10	5	5	–	–	–	Luceño *et al.* (1998)
		20	10	5	–	–	–	Luceño *et al.* (1998)
		20	10	5	1.04 ± 0.02	508.56	Antonina, PR	FUEL056037
		20	10	5	0.98 ± 0.05	479.22	Campo Largo, PR	FUEL055371
		20	10	5	1.25 ± 0.03	611.25	–	Ribeiro *et al.* (2018)
Pluriflorae	*R. albiceps* Kunth.	20	10	5	–	–	–	Vanzela *et al.* (2000)
	R. albobracteata A.C.Araújo	20	10	5	–	–	Carrancas, MG	FUEL055369
	R. consanguinea (Kunth.) Kükenth.	10	5	5	–	–	–	Vanzela *et al.* (2000)
	R. diamantina (C.B. Clarke) Kükenth.	24	12	6	–	–	–	Luceño *et al.* (1998)
	R. elatior Kunth	12	6	6	–	–	–	Ribeiro *et al.* (2018)
	R. globosa (Kunth) Roem. and Schult.	24	dysploid	5	–	–	–	Luceño *et al.* (1998)
		36	dysploid	5	–	–	Chapadão do Céu, GO	FUEL056063
		36	dysploid	5	–	–	–	Arguelho *et al*. (2012)
		37	dysploid	5	–	–	–	Luceño *et al.* (1998)
		43	dysploid	5	6.63 ± 0.03	3,242.07	Jaguariaíva, PR	FUEL056055
		45	dysploid	5	–	–	–	Arguelho *et al*. (2012)

Table continuation....								
		48	dysploid	5	–	–	–	Luceño *et al.* (1998)
		48	dysploid	5	–	–	Chapada dos Guimarães, MT	FUEL056126
		49	dysploid	5	9.16 ± 0.03	4,479.24	Tibagi, PR	FUEL056056
		50	dysploid	5	–	–	–	Ribeiro *et al.* (2018)
		58	dysploid	5	–	–	–	Arguelho *et al*. (2012)
		61	dysploid	5	11.32 ± 0.05	5,535.5	Carrancas, MG	FUEL056057
	R. riedeliana C.B. Clarke	12	6	6	–	–	–	Vanzela *et al.* (2000)
	R. rubra (Lour.) Makino	20	10	5	–	–	–	Hoshino (1987*b*)
	R. speciosa (Kunth.) Kükenth.	20	10	5	–	–	–	Vanzela *et al.* (2000)
	R. terminalis var. *rosemariana* (D. A. Simpson)	10	5	5	0.81 ± 0.01	396.09	Tibagi, PR	FUEL056058
		50	25	5	–	–	–	Luceño *et al.* (1998)
	R. warmingii Boeck.	*c.* 30	–	5	–	–	–	Vanzela *et al.* (2000)
Pauciflorae	*R. armerioides* J. Presl and C. Presl.	10	5	5	–	–	–	Luceño *et al.* (1998)
Pauciflorae	*R. barbata* (Vahl) Kunth	10	5	5	–	–	–	Vanzela *et al.* (2000)
		10	5	5	1.28 ± 0.02	625.92	Parque Guariba, PB	FUEL056054
Longirostres	*R. asperula*	18	9	9	–	–	–	Arguelho *et al*. (2012)
	R. corymbosa (L.) Britton	18	9	9	–	–	–	Nijalingapa *et al.* (1978)

Table continuation....

	18	9	9	–	–	–	Baquar (1978)
	18	9	9	–	–	–	Luceño *et al.* (1998)
	18	9	9	–	–	–	Arguelho *et al*. (2012)
	18	9	9	0.77 ± 0.02	376.53	Prado Ferreira, PR	FUEL056053
R. gigantea Link	18	9	9	–	–	–	Luceño *et al.* (1998)
	18	9	9	–	–	–	Arguelho *et al*. (2012)
R. legrandii Kükenth. ex Barros	18	9	9	–	–	–	Luceño *et al.* (1998)
R. pedersenii Guagl.	18	9	9	0.73 ± 0.04	356.97	Antonina, PR	FUEL056073
R. scutellata Griseb.	18	9	9	–	–	–	Luceño *et al.* (1998)
R. triflora Vahl	18	9	9	–	–	–	Luceño *et al.* (1998)

Note: Values preceded by *c.* have uncertain numbers of chromosomes.

Supplemental data 2. List of species of *Rhynchospora* and deposit numbers of the chloroplast sequences. Sequences were deposited in batches per species in the NCBI database. All genomes were made available by C. Buddenhagen (not yet published).

Taxa	GenBank accession/Submission ID
R. alba (L.) Vahl	2312461
R. albiceps Kunth.	2343194
R. barbata (Vahl) Kunth	2343202
R. barrosiana Guagl.	2343212
R. brasiliensis Boeck.	2343222
R. breviuscula H.Pfeiff.	2343226
R. cephalotes (L.) Vahl	2343232
R. chinensis Böckeler	2343237
R. nervosa subsp. *ciliata* T.Koyama	2343297
R. ciliolata Boeck.	2343339
R. colorata (L.) Hitchc.	2343342
R. comata (L.) Roemer and Schultes	2343344
R. consanguinea (Kunth.) Kükenth.	2343347
R. corymbosa (L.) Britton	2343799
R. emaciata (Nees) Boeck.	2343804
R. exaltata Kunth	2343811
R. eximia (Nees) Boeck.	2343819
R. fusca (L.) Aiton f.	2343823
R. gigantea Link	2343828
R. glaziovii Boeckeler	2343846
R. globosa (Kunth) Roem. and Schult.	2343861
R. holoschoenoides (Rich.) Herter	2343865
R. marisculus Lindl. and Nees	2343868
R. microcarpa Baldw. ex A. Gray	2343872
R. pubera (Vahl) Boeckeler	2343874
R. radicans (Schltdl. and Cham.) H. Pfeiff.	2343882
R. riedeliana C.B. Clarke	2343883
R. riparia (Nees) Boeckeler	2343904
R. robusta (Kunth.) Boeck	2343906

Taxa	GenBank accession/Submission ID
R. rubra (Lour.) Makino	2343909
R. rugosa (Vahl) Gale	2343912
R. speciosa (Kunth.) Kükenth.	2343916
R. tenerrima Nees ex Spreng.	2343919
R. tenuis Link	2343923
R. wightiana (Nees) Steudel	2343929

Supplementary data 3. Chloroplast coding sequences used and the percentage of pairwise residues that are identical in the alignment.

cpDNA genes	% Pairwise identity
atpA	95.9
atpB	95.8
atpE	95.2
atpF	94.5
atpH	97.0
atpI	96.7
cemA	94.9
matK	88.7
ndhC	96.6
ndhD	95.0
ndhE	93.5
ndhF	93.5
ndhG	97.7
ndhJ	95.8
ndhK	95.2
petA	96.2
petB	97.5
petD	97.4
petG	97.2
petL	95.0
petN	97.4
psaA	97.1

cpDNA genes	% Pairwise identity
psaB	97.2
psaC	96.9
psaI	96.9
psaJ	96.4
psbA	97.7
psbB	97.4
psbC	97.7
psbD	97.9
psbE	97.9
psbF	98.4
psbH	96.5
psbI	96.0
psbJ	97.3
psbK	97.5
psbL	98.4
psbM	98.5
psbN	97.1
psbT	97.0
psbZ	97.2
rbcL	96.7
rpl14	95.8
rpl16	95.6
rpl22	93.2
rpl36	94.2
rpoB	94.3
rpoC1	92.7
rpoC2	88.4
rps2	92.4
rps8	94.4
rps14	93.2
ycf3	96.1
ycf4	94.8

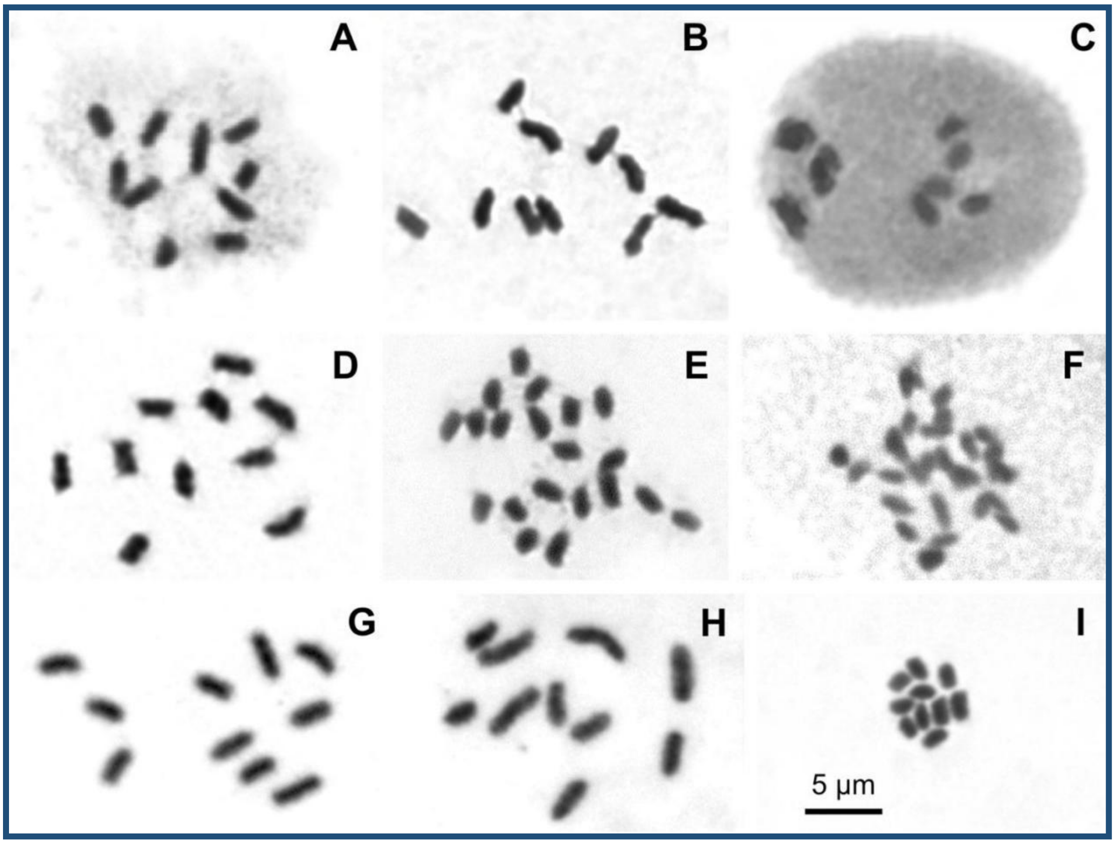

Supplemental data 4. Mitotic chromosomes of *Rhynchospora* sect. *Dichromena.* Metaphase of *R. breviuscula* 2*n* = 10 **(A)**. Metaphase of *R. nervosa* with 2*n* = 10 from Chapada dos Guimarães **(B)**. Pseudomonad of *R. nervosa* showing the degenerative domain (left) and the functional domain (right), which is at metaphase of pollen mitosis (*n* = 5) **(C)**. Metaphase of *R. nervosa* with 2*n* = 10 from Florianópolis **(D)**. Metaphase of *R. nervosa* with 2*n* = 20 **(E)**. Metaphase of *R. nervosa* with 2*n* = 30 from Cabo do Santo Agostinho **(F)**. Metaphase of *R. nervosa* subsp. *ciliata* with 2*n* = 10 **(G)**. Metaphase of *R. pubera* with 2*n* = 10 **(H)**. Metaphase of *R. setigera* with 2*n* = 10 **(I)**. All species present the same basic chromosome number *x* = 5, similar karyotypes in simmetry, and chromosome sizes decrease as chromosome numbers increase. Note that *R. setigera* has the smallest chromosomes, while *R. pubera* has the largest ones. Three ploidy levels are represented for *R. nervosa* **(B–F)**.

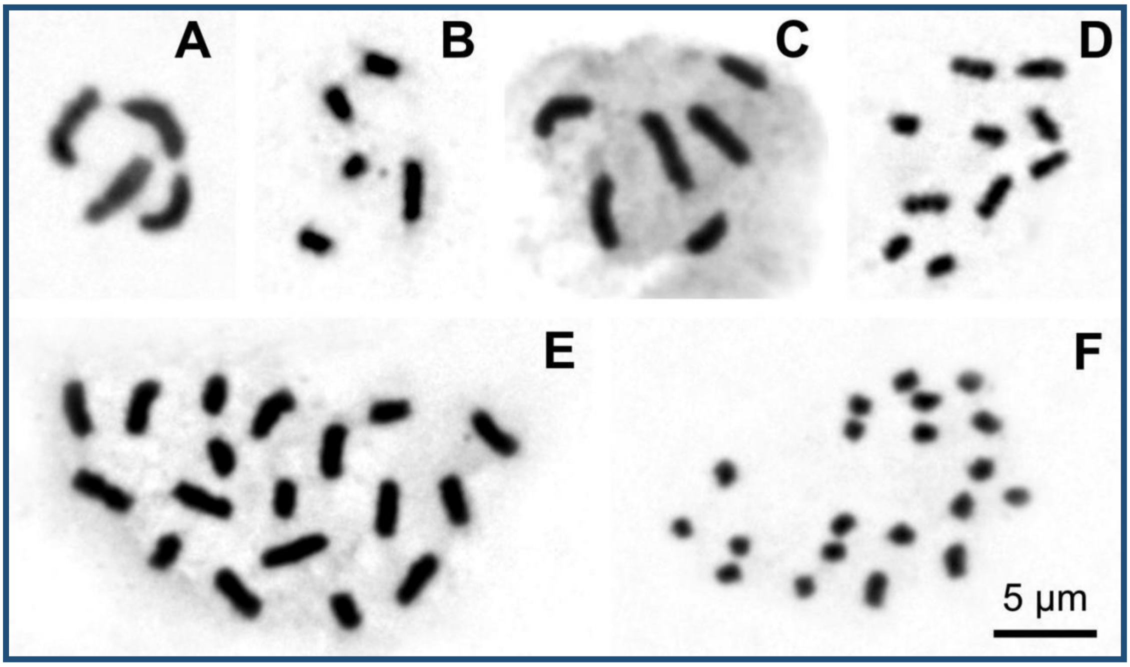

Supplemental data 5. Mitotic chromosomes of *Rhynchospora* sect. *Tenues* **(A–E)** and sect. *Spermodontes* **(F)**. Prometaphase of *R. tenuis* (2*n* = 4) **(A)**. Metaphase of *R. tenuis* (2*n* = 5) **(B)**. Prometaphases of *R. tenuis* (2*n* = 6) **(C)**. Metaphase of *R. riparia* (2*n* = 10) **(D)**. Metaphase of *R. tenuis* subsp. *austrobrasiliensis* (2*n* = 18) **(E)**. Metaphase of *R. tenerrima* (2*n* = 20) **(F)**. Note that sect. *Tenues* spp. display a variety of chromosome numbers, including dysploid karyotypes **(A–C)**, dysploid associated with polyploidy **(E)** and the polyploid *R. tenerrima* **(F)** with much smaller chromosomes than other species from this section.

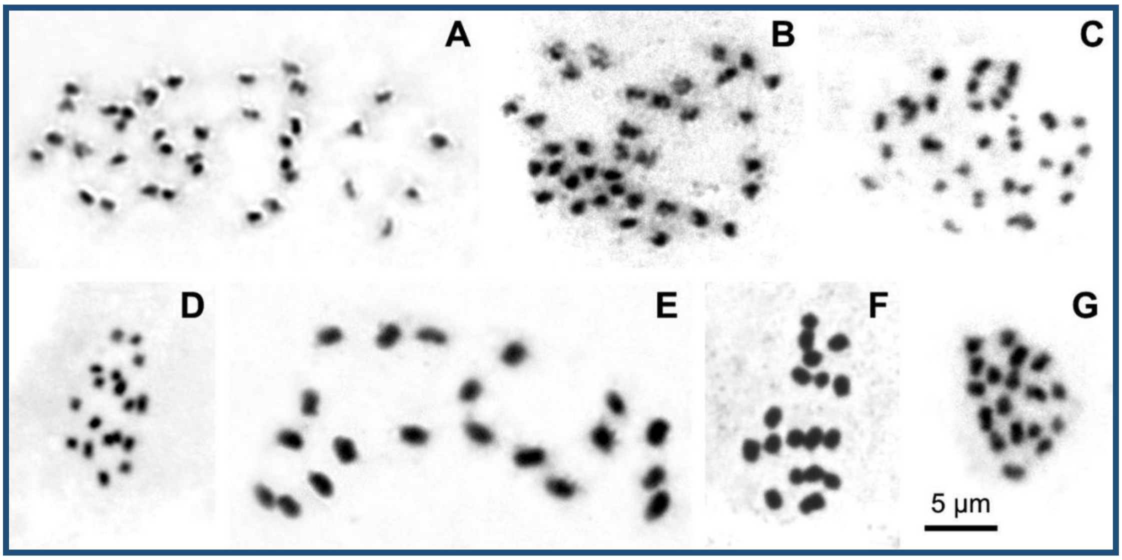

Supplemental data 6. Mitotic chromosomes of polyploids from four sections of *Rhynchospora* are shown: sect. *Glaucae* **(A–C)**, sect. *Cephalotae* **(D)**, sect. *Polycephalae* **(E)**, and sect. *Longirostres* **(F–G)**. Prometaphases of *R. rugosa, R. marisculus* and *R. dissitispicula* (respectively) from sect. *Glaucae*, all with 2*n* = 36 **(A–C)**. Metaphase of *R. cephalotes* (sect. *Cephalotae*) with 2*n* = 18 **(D)**. Metaphase of *R. holoschoenoides* (sect. *Polycephalae*) with 2*n* = 20 **(E)**. Metaphase of *R. corymbosa* and prometaphase of *R. pedersenii* (sect. *Longirostres*), both with 2*n* = 18 **(F–G)**. Three basic chromosome numbers are represented in this picture: *x* = 6 **(A–C)**, *x* = 9 **(D, F–G)** and *x* = 5 **(D)**. Higher ploidy species, from sect. *Glaucae* **(A–C)**, and *R. cephalotes* **(D)** have considerably smaller chromosomes compared to any other *Rhynchospora* spp. shown herein.

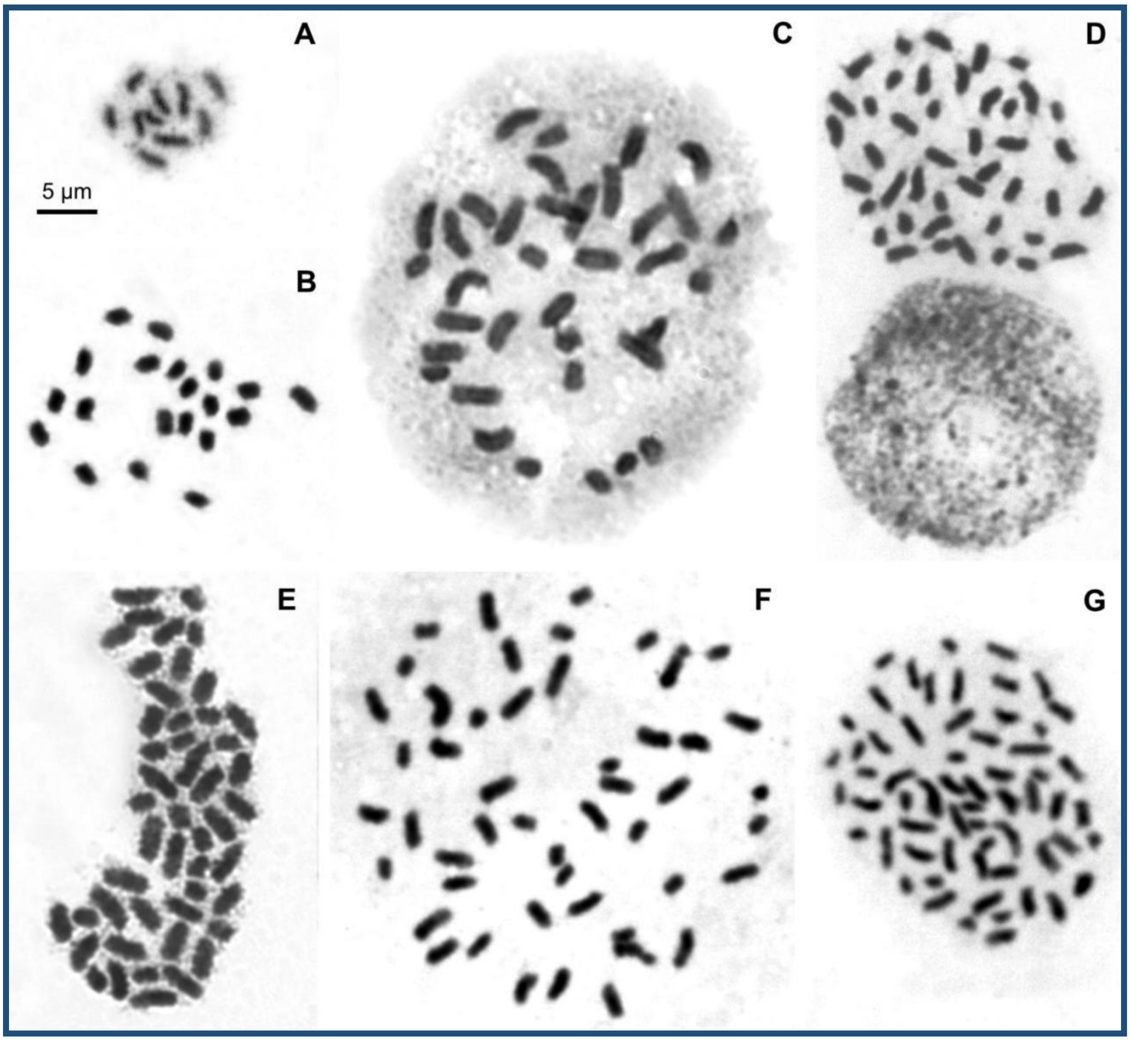

Supplemental data 7. Mitotic chromosomes of *Rhynchospora* sect. *Pluriflorae*. Prometaphase of *R. terminalis* var. *rosemariana* with 2*n* = 10 **(A)**. Metaphase of *R. albobracteata* with 2*n* = 20 **(B)**. Metaphase of *R. globosa* with 2*n* = 36 **(C)**. Metaphase (top) of *R. globosa* with 2*n* = 43, and interphase nucleus (bottom) showing numerous chromocenters **(D)**. Metaphase of *R. globosa* with 2*n* = 48 **(E)**. Metaphase of *R. globosa* with 2*n* = 49 **(F)**. Prometaphase of *R. globosa* with 2*n* = 61 **(G)**. Species from this section have chromosome numbers derived from *x* = 5 with *R. globosa* **(C–G)** presenting dysploidy associated with polyploidy. *Rhynchospora globosa* stands out as having the most diverse karyotype regarding chromosome number and size within a chromosome complement.

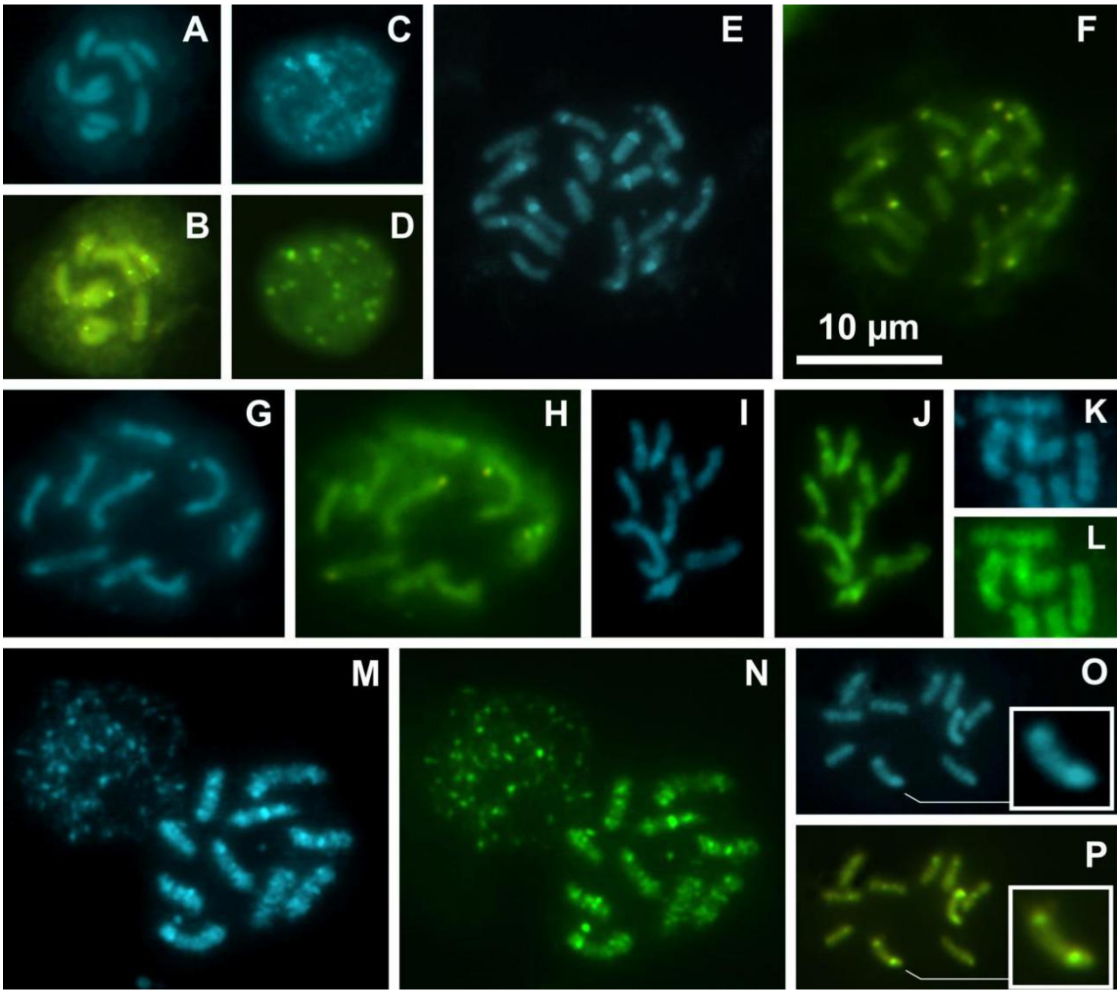

Supplemental data 8. C-CMA/DAPI banding in mitotic chromosomes and interphase nuclei of *Rhynchospora* sect. *Dichromena*. Prometaphase in *R. breviuscula* (2*n* = 10) with either $DAPI^0/CMA^+$ or $DAPI^-/CMA^+$ bands **(A–B)**. Nucleus of *R. breviuscula* with $DAPI^+/CMA^+$ chromocenters **(C–D)**. Prometaphases in *R. nervosa* from Carrancas with 2*n* = 20 **(E–F),** from Florianópolis with 2*n* = 10 **(G–H)**, and from Chapada dos Guimarães, with 2*n* = 10 **(I–J)**. CMA and DAPI heterochromatin are differently accumulated among chromosomes of *R. nervosa* from Chapada dos Guimarães **(K–L)**, with chromosomes either exhibiting only $DAPI^+$ bands, terminal and interstitial $DAPI^+/CMA^+$ bands, one interstitial $DAPI^+/CMA^+$ band, or none at all. Prometaphase in *R. nervosa* subsp. *ciliata* (2*n* = 10) showing many $DAPI^+/CMA^+$ signals, also visible in the nucleus **(M–N)**. Prometaphase of *R. setigera* with $DAPI^+/CMA^+$ signals **(I–J)**. Metaphase and prometaphase, respectively, in *R. nervosa* (2*n* = 20) **(O–P)**. $DAPI^+$ bands are all interstitial **(O)** while CMA^+ bands are either interstitial or terminal **(P)**. One chromosome with two large blocks of $DAPI^+/CMA^+$ heterochromatin, one terminal and the other subterminal, is depicted in detail (box).

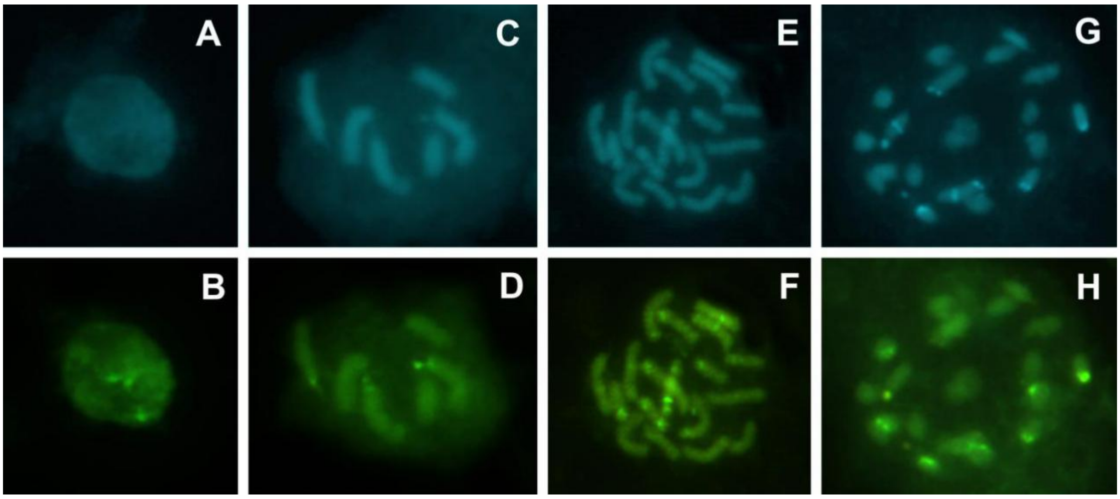

Supplemental data 9. C-CMA/DAPI banding in mitotic chromosomes and interphase nucleus of *Rhynchospora* sect. *Tenues* **(A–F)** and sect. *Spermodontes* **(G–H)**. Nucleus and prometaphase of *R. tenuis* with 2*n* = 6 showing the absence of $DAPI^+$ signals, and only three terminal CMA^+ ones **(A–D)**. Metaphase in *R. tenuis* subsp. *austrobrasiliensis* (2*n* = 18) showing CMA^+ signals **(E–F)**. Note the presence of several CMA^+ bands distributed throughout one chromosome. Prometaphase in *R. tenerrima* (2*n* = 20) with $DAPI^+/CMA^+$ and $DAPI^+/CMA^0$ blocks **(G–H)**.

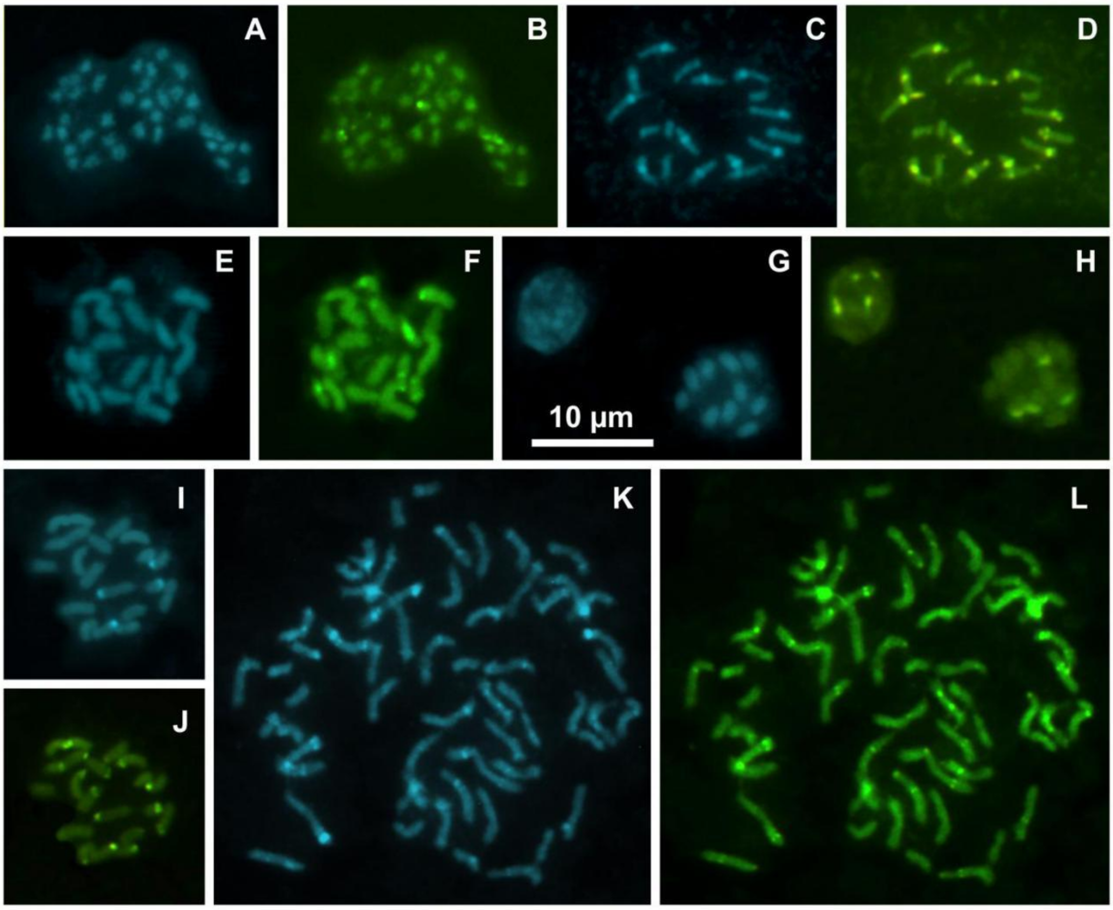

Supplemental data 10. C-CMA/DAPI banding in mitotic chromosomes and interphase nuclei of *Rhynchospora*. Prometaphase of *R. rugosa* (2*n* = 36) showing almost undetectable DAPI$^+$ spots, and four bright terminal CMA$^+$ signals **(A–B)**. Prometaphase of *R. corymbosa.* Note the large interstitial DAPI$^+$/CMA$^+$ blocks in several chromosomes, and some smaller terminal DAPI^0/CMA$^+$ ones **(C–D)**. Prometaphase of *R. holoschoenoides* with no DAPI$^+$ signals and some terminal and interstitial CMA$^+$ blocks **(E–F)**. Metaphasic chromosomes and interphasic nucleus of *R. terminalis* var. *rosemariana* showing four CMA$^+$/DAPI$^-$ signals, terminally located on the chromosomes **(G–H)**. Metaphase of *R. albobracteata*, with many terminal CMA$^+$ and fewer terminal DAPI$^+$ bands **(I–J)**. Prometaphasic chromosomes of *R. globosa* with 2*n* = 61 with several interstitial CMA$^+$/DAPI$^+$, some interstitial and terminal CMA$^+$, and some interstitial and terminal DAPI$^+$ bands **(K–L)**.

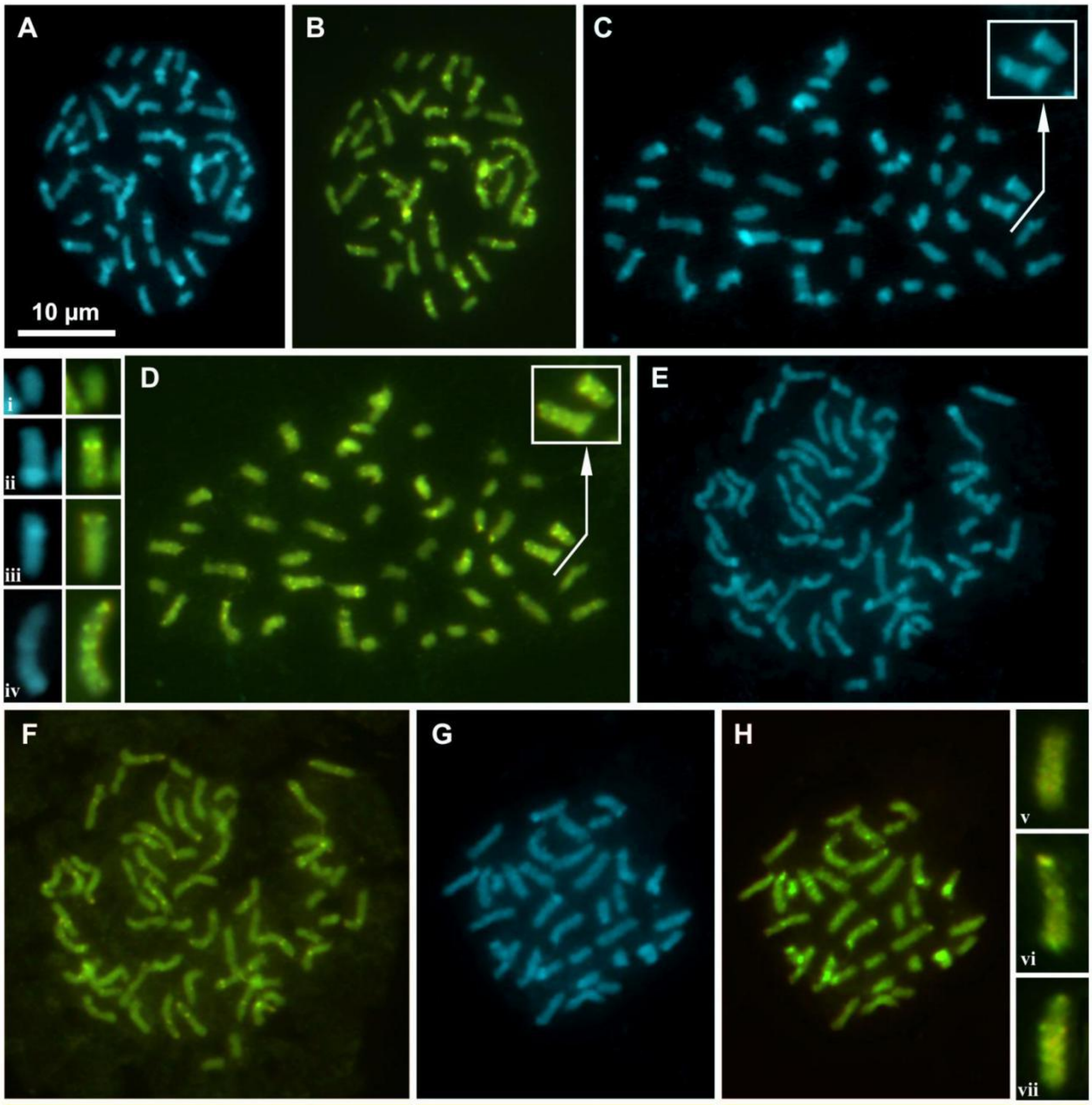

Supplemental data 11. C-CMA/DAPI banding in different populations of *Rhynchospora globosa*. Prometaphase with 2*n* = 43, from Jaguariaíva **(A–B)**. Chromosomes with different CMA/DAPI distribution are depicted **(i–iv)**. Metaphase with 2*n* = 49, from Tibagi **(C–D)**, showing two chromosomes with colocalized terminal CMA/DAPI bands and interstitial $CMA^{+}/DAPI^{0}$ bands (boxes). Prometaphase with 2*n* = 61, from Carrancas **(E–F)**. Prometaphase with 2*n* = 36, from Chapadão do Céu **(G–H)**. The individuals of this population have shown very faint $DAPI^{+}$ signals as opposed to many CMA^{+} ones. Three chromosomes with different CMA^{+} band distributions are represented in boxes **(v–vii)**. Note that, in three out populations, a unique large chromosome with many CMA^{+} bands stands out **(B**, **D**, **H**, **iv** and **vii)**.

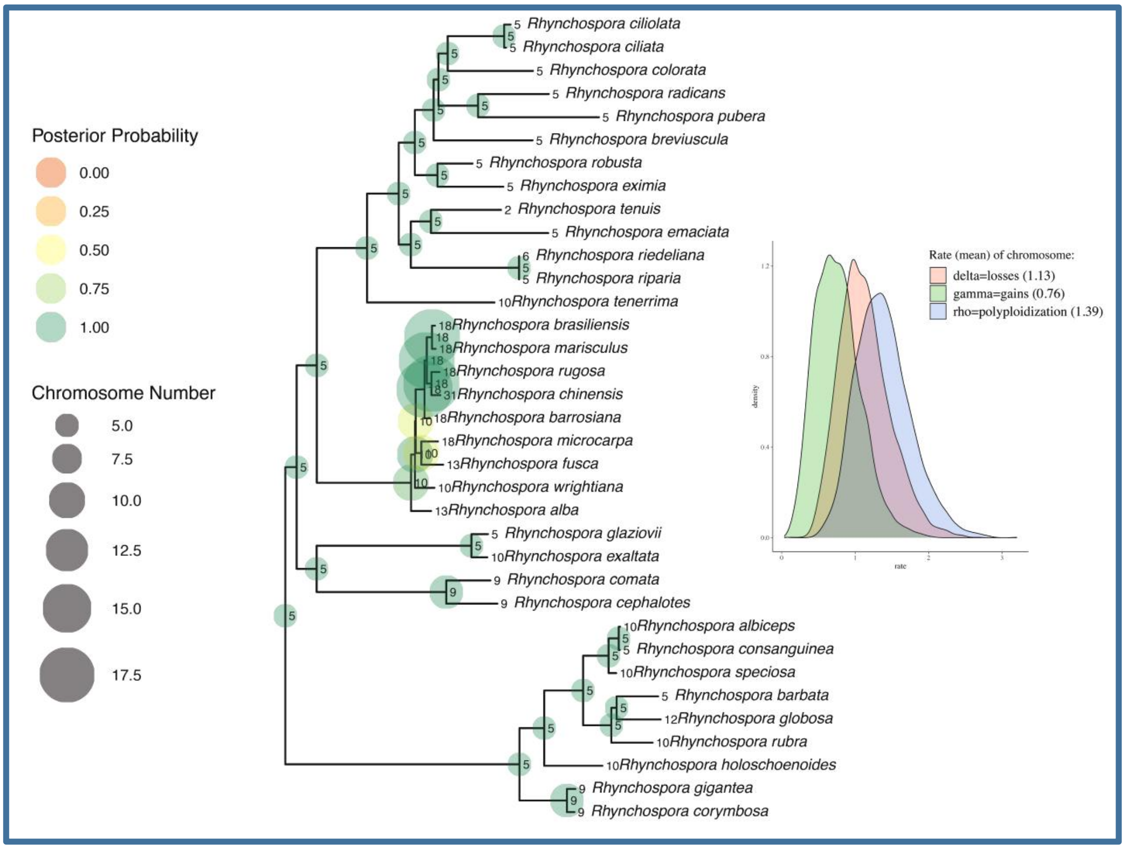

Supplemental data 12. RevBayes ChromEvol modelling of the ACN reconstruction along the Maximum Likelihood phylogenetic inference for Rhynchospora based on 54 chloroplast coding sequences (CDS) inferred with IQ-TREE, length: 51,383 bases. Density distributions show the estimated posterior values for the parameters rho, gamma and delta with their mean values in the legend.
